# Hypoxia-induced Nur77 activates PI3K/Akt signaling *via* suppression of Dicer/let-7i-5p to induce epithelial-to-mesenchymal transition

**DOI:** 10.7150/thno.52190

**Published:** 2021-01-19

**Authors:** Zeyu Shi, Sally K. Y. To, Shuaishuai Zhang, Shan Deng, Margarita Artemenko, Minda Zhang, Juan Tang, Jin-Zhang Zeng, Alice S.T. Wong

**Affiliations:** 1 School of Biological Sciences, University of Hong Kong, Pokfulam Road, Hong Kong.; 2 State Key Laboratory of Cellular Stress Biology and Fujian Provincial Key Laboratory of Innovative Drug Target Research, School of Pharmaceutical Sciences, Xiamen University, Xiamen, China.

**Keywords:** colorectal cancer, hypoxia, Nur77, microRNA biogenesis, PI3K/Akt, EMT

## Abstract

**Background:** Colorectal cancer (CRC) and the associated metastatic lesions are reported to be hypoxic. Hypoxia is a common feature in the tumor microenvironment and a potent stimulant of CRC. We have identified a regulatory role of Nur77 on Akt activation to enhance β-catenin signaling essential for CRC progression under hypoxic conditions.

**Methods:** The functional role of Nur77 in hypoxia-induced EMT was examined by scattering assays to monitor the morphologies of CRC cell lines under 1% O_2_. Sphere formation assays were performed to investigate whether Nur77 induced cancer stem cell-like properties in hypoxic CRC cells. The expression of various epithelial-to-mesenchymal transition (EMT) and stemness markers was analyzed by qPCR and Western blotting. Finally, Nur77 function and signaling *in vivo* was ascertained in subcutaneous tumor xenograft or liver metastasis model in nude mice using CRC cells stably transfected with appropriate constructs.

**Results:** Herein, we show, for the first time, that Nur77 is a novel regulator of microRNA biogenesis that may underlie its significant tumor-promoting activities in CRC cells under hypoxia. Mechanistically, Nur77 interacted with the tumor suppressor protein p63, leading to the inhibition of p63-dependent transcription of Dicer, an important miRNA processor and subsequent decrease in the biogenesis of let-7i-5p which targeted the 3'UTR of p110α mRNA and regulated its stability. Knockdown of Nur77 or overexpression of let-7i-5p inhibited the tumor metastasis *in vivo*.

**Conclusion:** Our data uncovered a novel mechanistic link connecting Nur77, Akt, and invasive properties of CRC in the hypoxic microenvironment.

## Introduction

Colorectal cancer (CRC) is the third most common cancer worldwide and accounts for ~10% of cancer-related mortality 1. The five-year overall survival rate for CRC patients with the early-stage disease is ~90%, but it significantly drops to ~10% for advanced-stage CRC 1. Therefore, new treatment strategies for these patients are highly warranted. Hypoxia is a common feature in the tumor microenvironment, and its severity varies between tumor types. On average, the intra-tumoral oxygen level is around 1-2% or below 2. Overexpression of hypoxia-inducible factor-1α (HIF-1α), a master switch for cellular adaptation to hypoxia, has been detected in primary and metastatic CRC lesions 3.

Hypoxia was shown to be a potent stimulant of Nur77 expression, which is transcriptionally activated by HIF-1α 4-6. During cancer development, Nur77 (also called NR4A1, TR3, or NGFIB) acts as a unique nuclear receptor to regulate a variety of functions, including cell proliferation, apoptosis, and invasion 7. The high expression level of Nur77 is evident in CRC and is associated with tumor stage, distant metastases, and poor patient survival 8. Despite its clinical significance, the oncogenic functions of Nur77 and the underlying molecular events remain largely unexplored. Our previous study has revealed a regulatory role of Nur77 on Akt activation to enhance β-catenin signaling under hypoxic conditions 6, yet the mechanism by which Nur77 regulates Akt remains unknown. Akt is a major downstream effector of the phosphatidylinositol 3-kinase (PI3K), a heterodimeric lipid kinase consisting of a regulatory subunit and a catalytic subunit. The class IA PI3K catalytic subunit encoded by the PIK3CA gene, p110α, is ubiquitously expressed in mammalian tissues. Upon activation by membrane receptors, p110α catalyzes the phosphorylation of phosphatidylinositol 4,5-bisphosphate (PIP2) to form phosphatidylinositol-3, 4, 5-triphosphate (PIP3), which acts as a docking site for the recruitment of kinases containing pleckstrin-homology (PH) domains, such as PDK1 and Akt 9.

In this study, we show, for the first time, that Nur77 plays a critical role in regulating microRNA (miRNA) biogenesis during hypoxia-induced epithelial-to-mesenchymal transition (EMT), a key metastatic process for cancer cells to acquire a more malignant phenotype for migration and invasion. Our results provide evidence that Nur77 promotes Akt activity through up-regulation of p110α expression *via* inhibition of Dicer-let-7i-5p under hypoxia.

## Methods

### Cell lines and cell culture

Human CRC cell line HCT116 was a gift from Dr. B. Vogelstein (Johns Hopkins Oncology Center, Baltimore, MD), and SW480 was obtained from American Type Culture Collection. HCT116 was grown in RPMI-1640 medium (Gibco, NY, USA), and SW480 was grown in DMEM (Gibco) at 37°C with 5% CO_2_. Media were supplemented with 100 units/mL penicillin, 100 μg/mL streptomycin and 5% fetal bovine serum. For hypoxic conditions, the cells were grown at 1% O_2_ in a modulator.

### Constructs and transient transfection

Cells were transfected with 0.25 μg plasmid DNA per well in 24-well plates for 24 h using Lipofectamine 2000 (Invitrogen, Carlsbad, CA, USA), followed by hypoxia incubation. Constructs encoding Nur77 and Nur77ΔDBD were previously described 6. The putative let-7i-5p binding site at 3'UTR of p110α (PIK3CA, nucleotide (nt) 7369 -7575 (207 bp), accession number NM_006218) was cloned into pcDNA3.1 vector with a luciferase insert driven by the CMV promoter. Luciferase reporter constructs containing the Dicer promoter were purchased from Addgene (pGL3-Dicer-prom, #25851). Mutant Dicer promoters were generated by site-directed mutagenesis and cloned using KpnI and HindIII restriction enzymes.

### Small interfering RNA, miRNA mimic, and miRNA inhibitor transfection

Small interfering RNA (siRNA) targeting Nur77 (SMARTpool), Dicer (SMARTpool), p63 (SMARTpool), and an NS duplex oligo (5'-GGC TAC GTC CAG GAG CGC A-3') were purchased from Dharmacon (Lafayette, CO, USA). Let-7i-5p, non-specific miRNA mimic and let-7i-5p inhibitor were purchased from Ambion (Austin, TX, USA). Cells were transfected with 20 nM siRNA, miRNA mimic or miRNA inhibitor using siLectFect (Bio-Rad, Hercules, CA, USA) following the manufacturer's instructions.

### miRNA identification

To identify the miRNA that is regulated by p110α on the hypoxic colon cancer cells, three prediction softwares (miRanda, TargetScan and RNAhybrid) were used. The analysis revealed 15 miRNAs (let-7f-3p, let-7i-5p, miR-132, miR-320a, miR-320b, miR-320d, miR-320c, miR-339-5p, miR-370, miR-422a, miR-520c-3p, miR-548, miR-520d-3p, miR-525-5p, and miR526b-3p) that have potential binding sites at the 3'UTR of p110α mRNA.

### Real-time polymerase chain reaction (qPCR)

Total RNA was extracted with Trizol Reagent (Invitrogen) and reverse transcribed into cDNAs using the M-MLV reverse transcriptase (Invitrogen). qPCR was done by AceQ qPCR SYBR Green Master Mix (High ROX Premixed) Kit (Vazyme, Nanjing, China) following the manufacturer's instructions. For precursor (pre-) and primary precursor (pri-) miRNA levels, immature miRNA was reversed-transcribed to cDNA by RevertAid First Strand cDNA Synthesis Kit (ThermoFisher) using random hexamer primer. The following specific primers were used: Nur77: forward: 5'-CCC TGA AGT TGT TCC CCT CAC-3' and reverse: 5'-GCC CTC AAG GTG TGG AGA AG-3'; E-cadherin: forward: 5'-ATT TTT CCC TCG ACA CCC GAT-3' and reverse: 5'-TCC CAG GCG TAG ACC AAG A-3'; N-cadherin: forward: 5'-AGC CAA CCT TAA CTG AGG AGT-3' and reverse: 5'-GGC AAG TTG ATT GGA GGG ATG-3'; Snail: forward: 5'-TCG GAA GCC TAA CTA CAG CGA-3' and reverse: 5'-AGA TGA GCA TTG GCA GCG AG-3'; Slug: forward: 5'-CGA ACT GGA CAC ACA TAC AGT G-3' and reverse: 5'-CTG AGG ATC TCT GGT TGT GGT-3'; CD133: forward: 5'-AGT CGG AAA CTG GCA GAT AGC-3' and reverse: 5'-GGT AGT GTT GTA CTG GGC CAA T-3'; CD44: forward: 5'-CTG CCG CTT TGC AGG TGT A-3' and reverse: 5'-CAT TGT GGG CAA GGT GCT ATT-3'; ALDH1: forward: 5'-CTG CTG GCG ACA ATG GAG T-3' and reverse: 5'-CGC AAT GTT TTG ATG CAG CCT-3'; EpCAM : forward: 5'-TGA TCC TGA CTG CGA TGA GAG-3' and reverse: 5'-CTT GTC TGT TCT TCT GAC CCC-3'; p110α: forward: 5'-CCA CGA CCA TCA TCA GGT GAA-3' and reverse: 5'-CCT CAC GGA GGC ATT CTA AAG T-3'; Dicer: forward: 5'-TGC TAT GTC GCC TTG AAT GTT-3' and reverse: 5'-AAT TTC TCG ATA GGG GTG GTC TA-3'; Drosha: forward: 5'-TGG CCA TGA CTC ATC CAA GT-3' and reverse: 5'-AGG GAG TTG GGT CAT CTT GG-3'; p63: forward: 5'-AAA GAC ATG CCC CAT CCA GA-3' and reverse:5'- CAT ACT GGG CAT GGC TGT TC-3'. Dicer promoter primer AB: forward: 5'-AAA TCA GAC AAA CCG GCC AG-3' and reverse: 5'-ATC TCG TCT CAC TGC AAC CT-3'; Dicer promoter primer C: forward: 5'- GGC GAC AAG AGT GAA ACT CT-3' and reverse: 5'- ATC TCA GTA TGG GGG TAG GC-3'; GAPDH: forward: 5'-GGA GCG AGA TCC CTC CAA AAT-3' and reverse: 5'- GGC TGT TGT CAT ACT TCT CAT GG-3'. Immature miRNA levels were amplified by qPCR using specific primers as follows: pre-let-7i: forward: 5'-TGT GCT GTT GGT CGG GT-3' and reverse: 5'- GCA GTA GCT TGC GCA GTT-3'; pri-let-7i: forward: 5'- CGA GGA AGG ACG GAG GA -3' and reverse: 5'-GCT GAG CAT CAC CAG CAC-3'; pre-U6: forward: 5'-CTC GCT TCG GCA GCA CA-3' and reverse: 5'-AAC GCT TCA CGA ATT TGC GT-3'. To detect miRNA levels, miRNA was first reversed-transcribed to cDNA by TaqMan miRNA reverse transcription kit (Applied Biosystems, CA, USA) using specific stem-loop primers let-7i-5p: 5'-GTC GTA TCC AGT GCA GGG TCC GAG GTA TTC GCA CTG GAT ACG AAA CAG C-3'; U6 small nuclear RNA (internal control): 5'-GTC GTA TCC AGT GCA GGG TCC GAG GTA TTC GCA CTG GAT ACG ACA AAA AAT-3'. miRNA levels were then amplified by qPCR using specific primers as follows: let-7i-5p: forward: 5'- TGA GGT AGT AGT TTG TGC TGT T-3'; U6: Forward: 5'-TTC CTC CGC AAG GAT GAC ACG C-3'; Universal reverse: 5'-GTG CAG GGT CCG AGG T-3'. Relative quantification was achieved by normalization to the amount of GAPDH using 2^-ΔΔCt^ method. Unspecific signals caused by primer dimers were excluded by non-template controls and dissociation curve analysis.

### Measurement of mRNA stability

To determine the half-life of p110α mRNA, cells transfected with NS or Nur77 siRNA were treated with actinomycin D (5 μg/mL) under hypoxia. Cells were harvested at various time points, and mRNA expression was quantified by qPCR as described above.

### Western blotting

Cells were lysed with SDS lysis buffer (10 mM Tris-HCl, 2 mM EDTA, 1% SDS). Equal amounts of cell lysates were separated by 7.5% sodium dodecyl sulfate-polyacrylamide gel electrophoresis. Proteins were electro-transferred to nitrocellulose membranes and incubated with primary antibodies against Snail (C15D3, CST, Danvers, MA, USA), Slug (C19G7, CST), E-cadherin (CST), N-cadherin (CST), CD44 (156-3C11, CST), CD133 (D2V8Q, CST), EpCAM (CST), ALDH1A1 (D9J7R, CST), p110α (CST), Nur77 (D63C5, CST), p-mTOR (S2448, CST), mTOR (7C10, CST), p-p70S6K (Thr389, CST), p70S6K (CST), p-4E-BP1 (Thr37/46, CST), 4E-BP1 (53H11, CST), p63 (D9L7L, CST), p53 (1C12, CST), Dicer1 (D38E7, CST), Drosha (D28B1, CST), phospho-Akt (Ser473, CST), total Akt (C67E7,CST) or β-actin (CST). For Western blotting, antibodies were used at the dilution of 1:1000. The membranes were incubated with appropriate horseradish peroxidase-conjugated secondary antibodies (Bio-Rad). The protein bands were visualized using enhanced chemiluminescence detection reagents (Amersham, Little Chalfont, UK). Densities of bands were measured using Image J. Relative quantification was achieved by normalization to the amount of β-actin.

### Co-immunoprecipitation

After incubation for 24 h under hypoxia or normoxia, HCT116 cells were lysed and sonicated in 500 μL IP lysis buffer containing 150 mM NaCl, 100 mM NaF, 50 mM Tris-HCl (pH 7.6), 0.5% NP-40 and 1 mM PMSF. The lysates were incubated with antibodies against Nur77 (D63C5, CST) or p63 (D9L7L, CST) and purified with protein A/G beads (Santa Cruz, California, USA) at 4 °C overnight. The eluted proteins were separated by 10% SDS-PAGE and detected with anti-Nur77, anti-p63, and anti-p53 antibodies.

### Luciferase reporter assay

For 3'UTR luciferase assays, HCT116 and SW480 cells were co-transfected with let-7i-5p or NS mimic and p110α-3'UTR luciferase reporter constructs using Lipofectamine 2000, following the manufacturer's instructions. Renilla luciferase-expressing plasmid was also co-transfected for normalizing transfection efficiency. For the Dicer-promoter reporter assays, HEK293T cells were transfected with Dicer reporter construct and Nur77 and/or p63 expression plasmid, and Renilla luciferase plasmid. To further test for endogenous activity of Nur77, HCT116 and SW480 cells were co-transfected with Nur77 or non-specific siRNA, Dicer promoter construct and Renilla luciferase plasmid. After transfection for 48 h, cells were lysed with reporter lysis buffer (Promega, San Luis Obispo, CA, USA). Luciferase activities were measured by VictorX4 multilabel plate reader (PerkinElmer, Waltham, MA, USA) after the addition of luciferase substrates (Promega), according to the manufacturer's instructions.

### Cell scattering assay

Cell scattering assays were performed to evaluate EMT morphologic changes. Briefly, 2000 cells were plated in six-well plates. When small colonies formed, cells were transfected with the indicated siRNA and incubated in the hypoxic chamber for 48 h. Scattered colonies were judged by a typical change in morphology characterized by cell-cell dissociation and the acquisition of a migratory fibroblast-like phenotype. Fifty colonies were randomly scored for each well under light microscope.

### Sphere formation assay

Cells were transfected with NS or Nur77 siRNA for 48 h; empty vector pcDNA3.1 or Nur77 constructs for 24 h. Enrichment of cancer stem cells was performed in serum-free RPMI-1640 and DMEM supplemented with 100 units/mL penicillin, 100 μg/mL streptomycin using non-tissue culture treated dished coated with 1% agarose. Cell were seeded at 5,000 cells/mL and cultured for 72 h under hypoxic condition till tumor spheres were formed. For serial spheroid formation, primary spheroids were dissociated and reseeded under the hypoxic condition to obtain secondary spheroids. Same procedures were repeated for the formation of tertiary spheroids.

### Chromatin immunoprecipitation (ChIP) assay

ChIP assay was performed with High-Sensitivity ChIP Kit (Abcam, Cambridge, UK) following manufacturer's instructions. Briefly, cells were lysed and the pellet containing chromatin was resuspended with 500 μl ChIP Buffer. The chromatin was then extracted by sonication shearing. The ChIP reaction mixtures were incubated at 4°C overnight, and the DNA was released and purified for the following PCR test.

### *In vivo* studies

All animal experiments were approved by the Institutional Animal Care and Use Committee at the University of Hong Kong. 2✕10^6^ HCT116 cells alone or stably transfected with non-specific (NS) shRNA or Nur77 shRNA were subcutaneously injected into the right flank of BALB/c nude mice. Body weight and tumor volume were measured and recorded every day after injection. The length and width of tumors were measured with a caliper, and tumor volume was calculated as (length+width^2^)/2. After removing unwanted surrounding tissues, tumor samples were either fixed with formalin for paraffin embedding or cut into small pieces and homogenized for protein extraction, followed by co-immunoprecipitation or Western blot analysis. Total RNA was extracted from the paraffin blocks using the RNeasy FFPE kit (Qiagen, Venlo, Netherlands) according to the manufacturer's instructions. For the liver metastasis model, HCT116 stably expressing non-specific or Nur77 shRNA were harvested from subconfluent cultures and washed once in PBS and resuspended in PBS. After the nude mice were anesthetized with pentobarbital sodium, a small left abdominal flank incision was made, and 5✕10^5^ of cells in 50 μL PBS were injected into the spleen parenchyma (n = 6/group, repeated three times). To avoid intrasplenic tumor growth, the spleen was removed after 10 minutes. After the mice were euthanized 6 weeks later, livers were removed, photographed and the numbers of metastatic nodules were counted.

### Immunohistochemistry

Paraffin sections from mice tumors were deparaffinized with xylene and rehydrated in graded ethanol. After blocking and heat-induced antigen retrieval using citrate buffer, the slides were separately incubated with antibodies against PCNA (Abcam, Cambridge, UK; 1:200), Nur77 (Abclonal, Woburn, MA; 1:200) and IgG (Abclonal, Woburn, MA; 1:200) overnight at 4 °C. After incubation with anti-rabbit or anti-mouse secondary antibodies (peroxidase-labeled) (1:200) for 2 h at room temperature, the slides were further detected with DAB Detection Kit (Abcam, Cambridge, UK).

### Statistical analysis

All experiments were repeated at least three times, and data were presented as mean ± S.D. Student's *t* test or two-way ANOVA were performed in GraphPad Prism (GraphPad, San Diego, CA) to determine the differences between the experimental groups. *P* < 0.05 is considered to be statistically significant.

## Results

### Nur77 is a critical regulator of hypoxia-induced morphological changes and cancer stem cell (CSC)-like phenotypes

**Figure 1:**
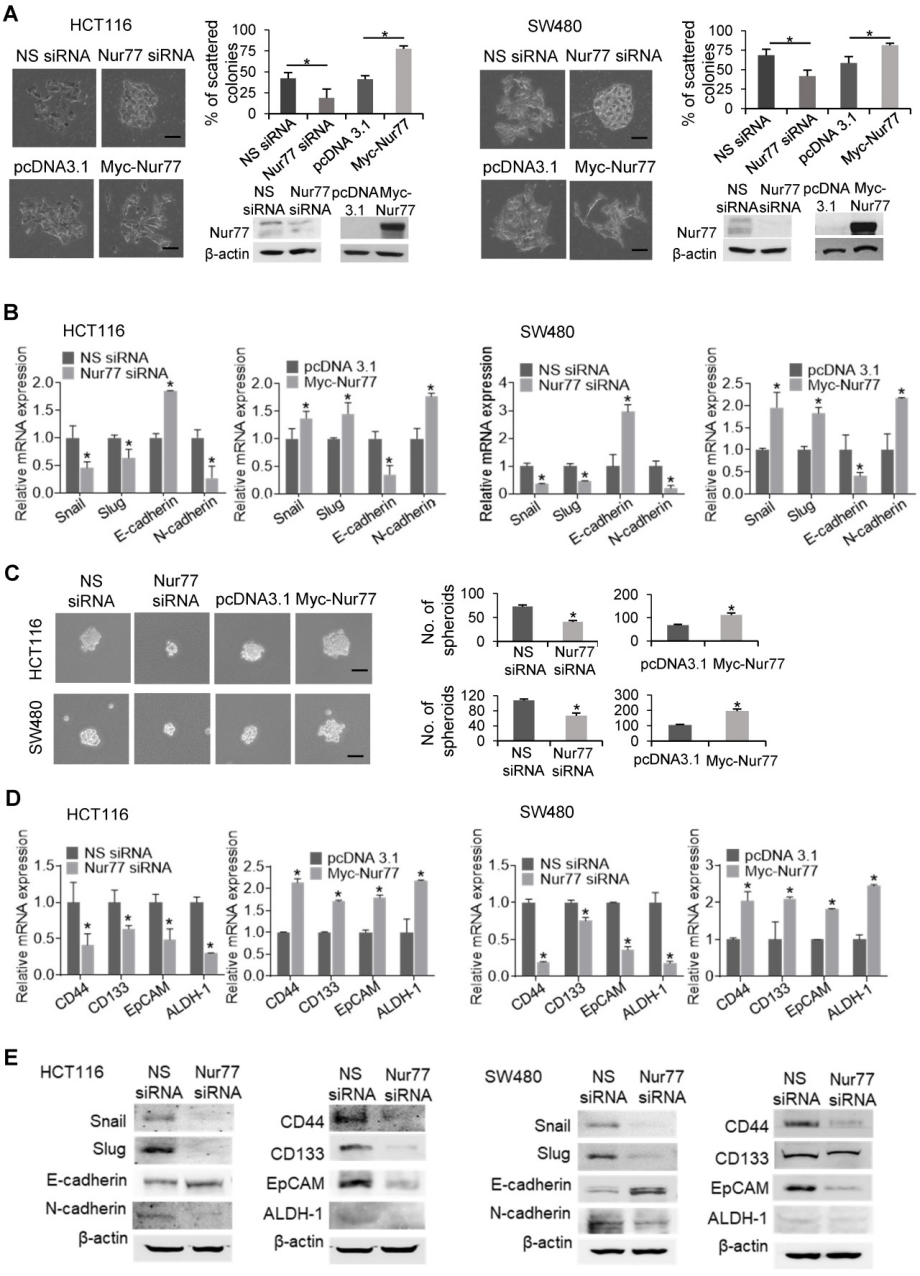
** Nur77 is a critical regulator of hypoxia-induced morphological changes and CSC-like phenotypes.** HCT116 and SW480 cells were transfected with non-specific (NS) siRNA or Nur77 siRNA for 48 h; empty vector pcDNA3.1 or Nur77 constructs for 24 h, followed by incubation at 1% O_2_.** (A)** The cells were fixed and observed for EMT morphologic changes. Representative scattered colonies were imaged and scattered colonies were scored from five random fields of view. Knockdown and overexpression of Nur77 were confirmed by Western blotting (inset). **(B)** Expression of EMT markers Snail, Slug, E-cadherin, and N-cadherin were determined by qPCR. **(C)** Transfected cells were allowed to form tumor spheres. Spheres were photographed (left panel) and counted (right panel). **(D)** Expression of colon cancer stem cell markers CD44, CD133, EpCAM, and ALDH-1 were detected by qPCR. GAPDH served as an internal control for qPCR. **(E)** Expression of EMT markers Snail, Slug, E-cadherin, and N-cadherin and CSC markers CD44, CD133, EpCAM, and ALDH-1 were detected by Western blots. For qPCR, relative quantification was achieved by normalization to the amount of GAPDH using 2^-ΔΔCt^ method. For Western blotting, the band intensities were quantified by densitometric analysis. Data is presented as mean ± SD., and one of three independent experiments is shown **(A)**, **(C)**, **(E)**, or as mean ± SD. of three biological replicates **(B)**, **(D)**; * indicates *P* < 0.05 in paired Student's *t* test (Nur77 siRNA compared with NS siRNA and Myc-Nur77 compared with pcDNA3.1). Bar, 50 µm.

To characterize the functional role of Nur77 in hypoxia-induced EMT, we first performed scattering assays with two CRC cell lines, HCT116 and SW480, under 1% O_2_. Cells transfected with Nur77 siRNA adopted epithelial-like phenotypes capable of forming compact colonies, whereas the growth of control cells was scattered (Figure 1A). Consistent with morphological changes, Nur77 siRNA resulted in up-regulation of E-cadherin (epithelial marker) and down-regulation of Snail, Slug, and N-cadherin (mesenchymal markers) (Figure 1B). Conversely, ectopic Nur77 expression resulted in more scattered colonies, inducing typical mesenchymal morphologies with corresponding changes of EMT genes (Figure 1A-B).

EMT is closely associated with the emergence of CSCs 10. To test whether Nur77 might confer CSC-like properties in hypoxic CRC cells, we performed the sphere formation assay. In Nur77 siRNA-treated cultures, smaller and fewer spheres were observed under hypoxia (Figure 1C), indicating that the sphere-forming ability of CRC cells was impaired by Nur77 knockdown. In contrast, overexpression of Nur77 promoted sphere formation (Figure 1C). These results were supported by qPCR analyses of four CSC markers: CD44, CD133, epithelial cell adhesion molecule (EpCAM) and aldehyde dehydrogenase 1 (ALDH-1), which are highly expressed in colorectal CSCs 11. These markers were markedly reduced by Nur77 knockdown but increased by overexpression of Nur77 (Figure 1D). Protein expression of EMT and CSC markers were also affected consistently with mRNA levels (Figure 1E). However, no apparent changes were found under normoxia (Figure S1A-B), indicating that the hypoxic microenvironment may strengthen the modulatory property of Nur77 on EMT and CSCs. We also conducted serial spheroid formation assays to more precisely examine Nur77 effects on CSC self-renewal capacity. Our results showed that both HCT116 and SW480 cells could strongly develop secondary and tertiary spheroids, a characteristic feature greatly impaired by Nur77 siRNA but enhanced by Nur77 overexpression (Figure S1C). Taken together, these results suggest that Nur77 may play an important role in modulating EMT and CSC-like phenotypes in hypoxic CRC.

### Hypoxia induces p110α expression and Akt/mTORC1 activation *via* Nur77

**Figure 2:**
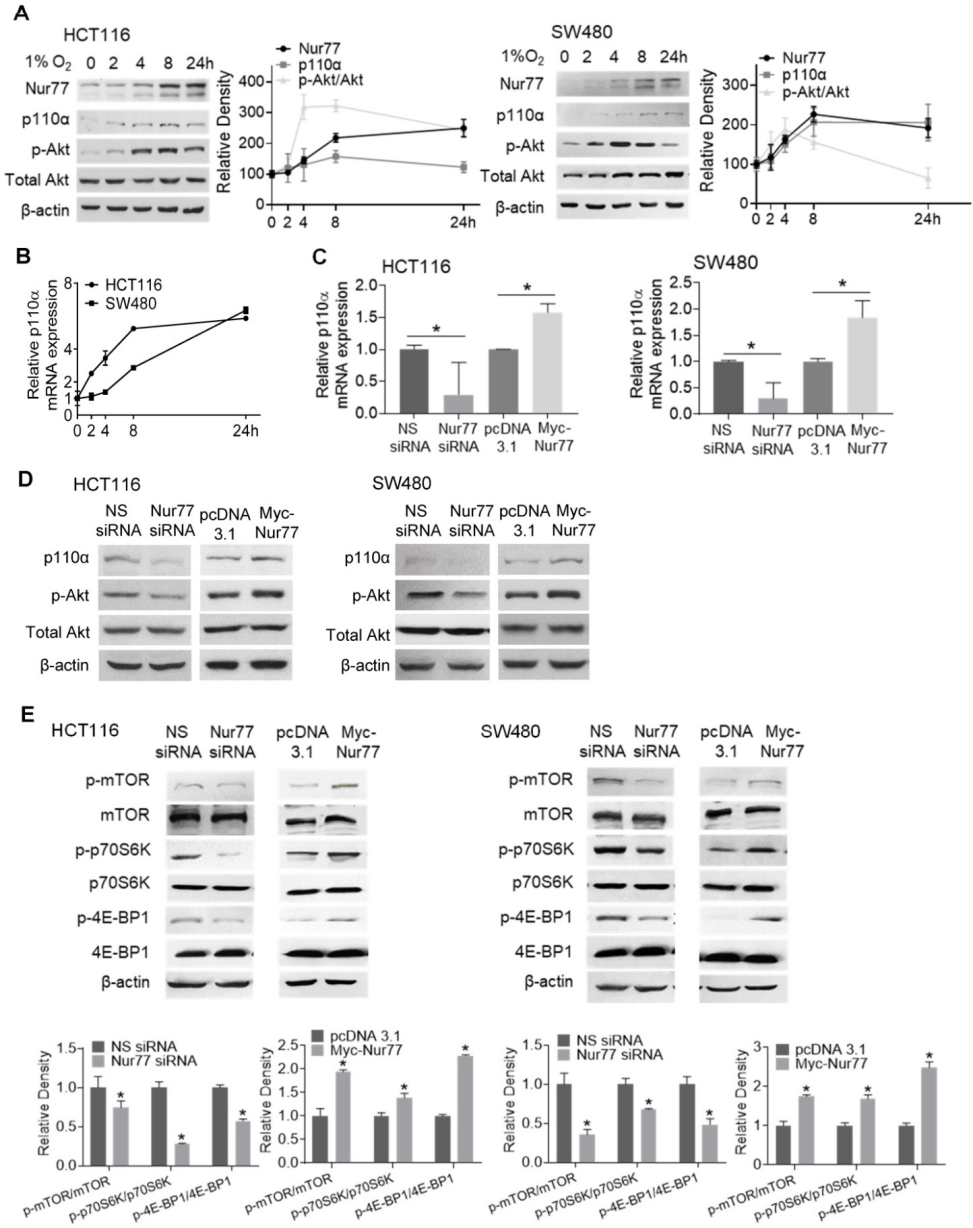
** Hypoxia induces p110α expression and Akt/mTORC1 activation *via* Nur77.** HCT116 and SW480 cells were incubated at 1% O_2_ for different time periods (0, 2, 4, 8 24 h). **(A)** Total proteins were extracted and analyzed by Western blotting to determine the expression levels of Nur77, p110α, p-Akt, and total Akt, and **(B)** qPCR was performed to determine the mRNA level of p110α. GAPDH was included as an internal control. HCT116 or SW480 cells were treated with non-specific (NS) siRNA, Nur77 siRNA, empty vector pcDNA3.1, or Nur77 construct, followed by 1% O_2_ incubation. **(C)** The mRNA level of p110α was determined by qPCR.** (D)** The protein levels of p110α, p-Akt, and total Akt were determined by Western blotting.** (E)** Phosphorylated and total levels of mTOR, p70S6K, and 4E-BP1 were analyzed by Western blotting. β-actin was included as an internal control. For qPCR, relative quantification was achieved by normalization to the amount of GAPDH using 2^-ΔΔCt^ method. For Western blotting, the band intensities were quantified by densitometric analysis. Data is presented as mean ± SD., and one of three independent experiments is shown **(A)**, **(D)**, **(E)**, or as mean ± SD. of three biological replicates **(B)**, **(C)**; * indicates *P* < 0.05 in paired Student's *t* test (Nur77 siRNA compared with NS siRNA and Myc-Nur77 compared with pcDNA3.1).

Hyperactivation of PI3K/Akt is critical for enhancing EMT and tumor metastasis 12. We previously reported that Nur77 is a key regulator of PI3K/Akt signaling under hypoxia 6. However, the mechanism by which hypoxia triggers Nur77-dependent Akt activity remains unknown. Since PI3K-p110α is an important kinase in regulating Akt activation, hypoxia's impact on p110α expression was determined. We harvested CRC cells incubated in the hypoxic chamber at various time points. Western blot analyses showed that hypoxia-induced Nur77 expression was paralleled by an increase in both p110α and phosphorylated Akt (p-Akt) (Figure 2A). We also found that the gradual increase in p110α protein levels corresponded to its mRNA expression, as detected by qPCR (Figure 2B). Since p-Akt levels sharply increased at around 4 h in both HCT116 and SW480 cells, we chose this time point for subsequent analyses unless otherwise specified.

We next investigated if p110α and p-Akt could be affected by Nur77 under the hypoxic condition. Nur77 siRNA efficiently blocked hypoxia-induced p110α expression at both mRNA and protein levels, and also Akt phosphorylation (Figure 2C-D), while overexpression of Nur77 exerted an opposite effect (Figure 2C-D), indicating that hypoxia activated p110α/Akt in a Nur77-dependent manner. Also, no significant differences were identified in normoxia (Figure S2A-B), further suggesting hypoxia as an important requisite for Nur77-regulated p110α/Akt activation. Since the mTORC1 complex is an important downstream mediator of Akt signaling to promote various growth effects, we investigated whether Nur77 could affect its functions. Indeed, Nur77 siRNA transfection decreased the phosphorylated mTOR level and concurrently, the phosphorylation of p70^S6K^ and 4E-BP1, two primary mTORC1 target proteins, was inhibited (Figure 2E). Overexpression of Nur77 increased mTORC1 phosphorylation and its downstream effectors (Figure 2E). These results suggested a significant impact of Nur77 on activating the oncogenic PI3K/Akt/mTORC1 signaling.

### Nur77 enhances p110α mRNA stability independent of its DNA binding ability

**Figure 3:**
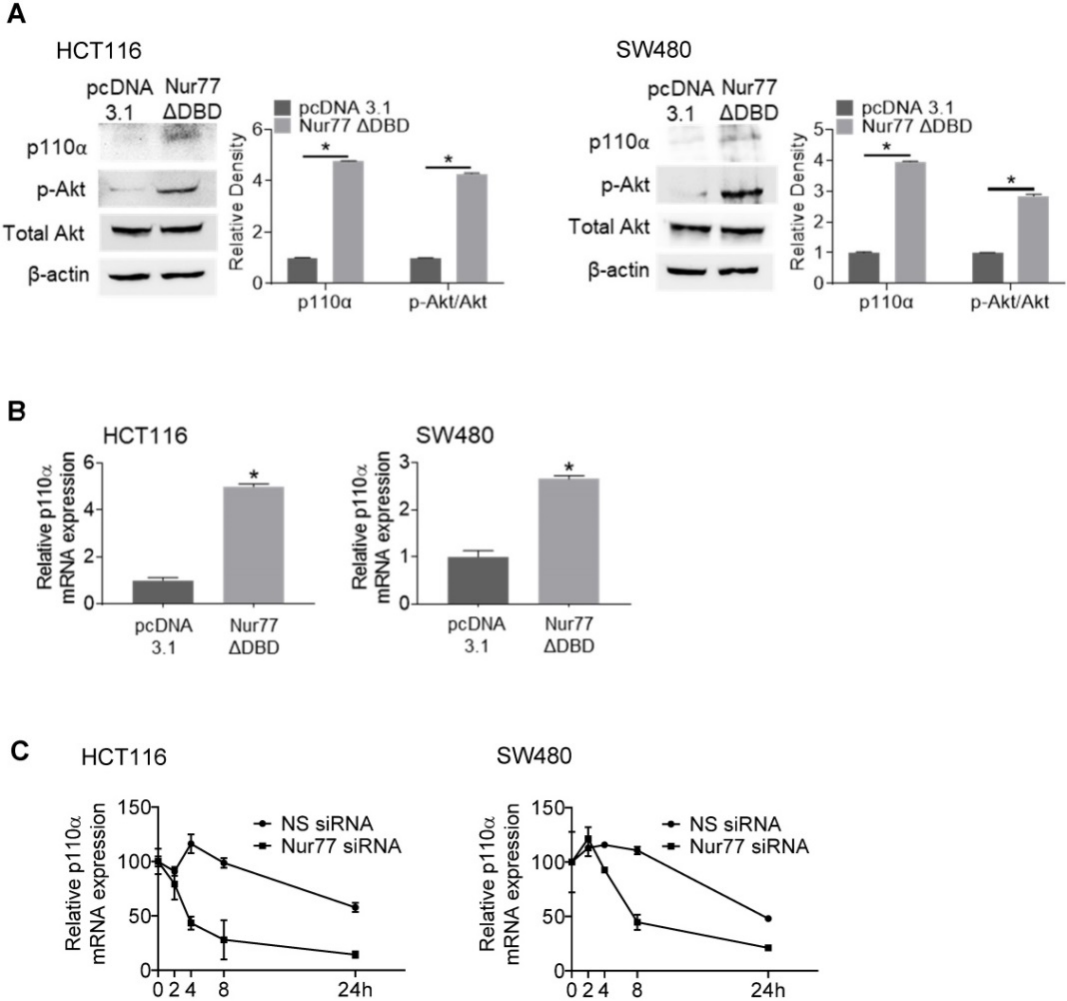
** Nur77 enhances p110α mRNA stability.** HCT116 or SW480 cells were transfected with empty vector pcDNA3.1 or Nur77 lacking the DNA-binding domain (Nur77ΔDBD) for 24 h, then incubated at 1% O_2_. **(Α)** Protein levels (p110α, p-Akt, and total Akt) and **(B)** p110α mRNA levels were detected by Western blotting and qPCR respectively. β-actin and GAPDH were included as an internal control.** (C)** HCT116 cells or SW480 cells transfected with non-specific (NS) or Nur77 siRNA were treated with actinomycin D (Act-D; 5 µg/mL) for different time periods (0, 2, 4, 8, and 24 h). qPCR was conducted to determine p110α expression level. GAPDH was included as an internal control. For qPCR, relative quantification was achieved by normalization to the amount of GAPDH using 2^-ΔΔCt^ method. For Western blotting, the band intensities were quantified by densitometric analysis. Data is presented as mean ± SD., and one of three independent experiments is shown **(A)**, or as mean ± SD. of three biological replicates **(B)**, **(C)**; * indicates *P* < 0.05 in paired Student's *t* test (Nur77ΔDBD compared with pcDNA3.1 and Nur77 siRNA compared with NS siRNA).

Interestingly, a Nur77 mutant lacking the DNA-binding domain (Nur77ΔDBD) could induce p110α mRNA and protein levels similar to wild-type Nur77 (Figure 3A-B), suggesting that Nur77 might regulate p110α expression independent of its DNA-binding activity. These results led us to propose that Nur77 might stabilize p110α mRNA by interfering with its degradation instead of transcription. Hence, we carried out RNA decay analysis using the RNA polymerase inhibitor actinomycin D. As shown in Figure 3C, p110α mRNA half-life in Nur77 siRNA-transfected cells was shortened by approximately 4-fold when compared with NS siRNA-transfected cells, supporting the idea that Nur77 could inhibit the degradation of p110α mRNA and enhance its stability. Under normoxia, Nur77 failed to enhance p110α mRNA stability in HCT116 and SW480 cells (Figure S3), suggesting that hypoxia could be an essential criterion for Nur77 to maintain its function.

### Nur77 inhibits Dicer and let-7i-5p to induce p110α expression and Akt phosphorylation

**Figure 4:**
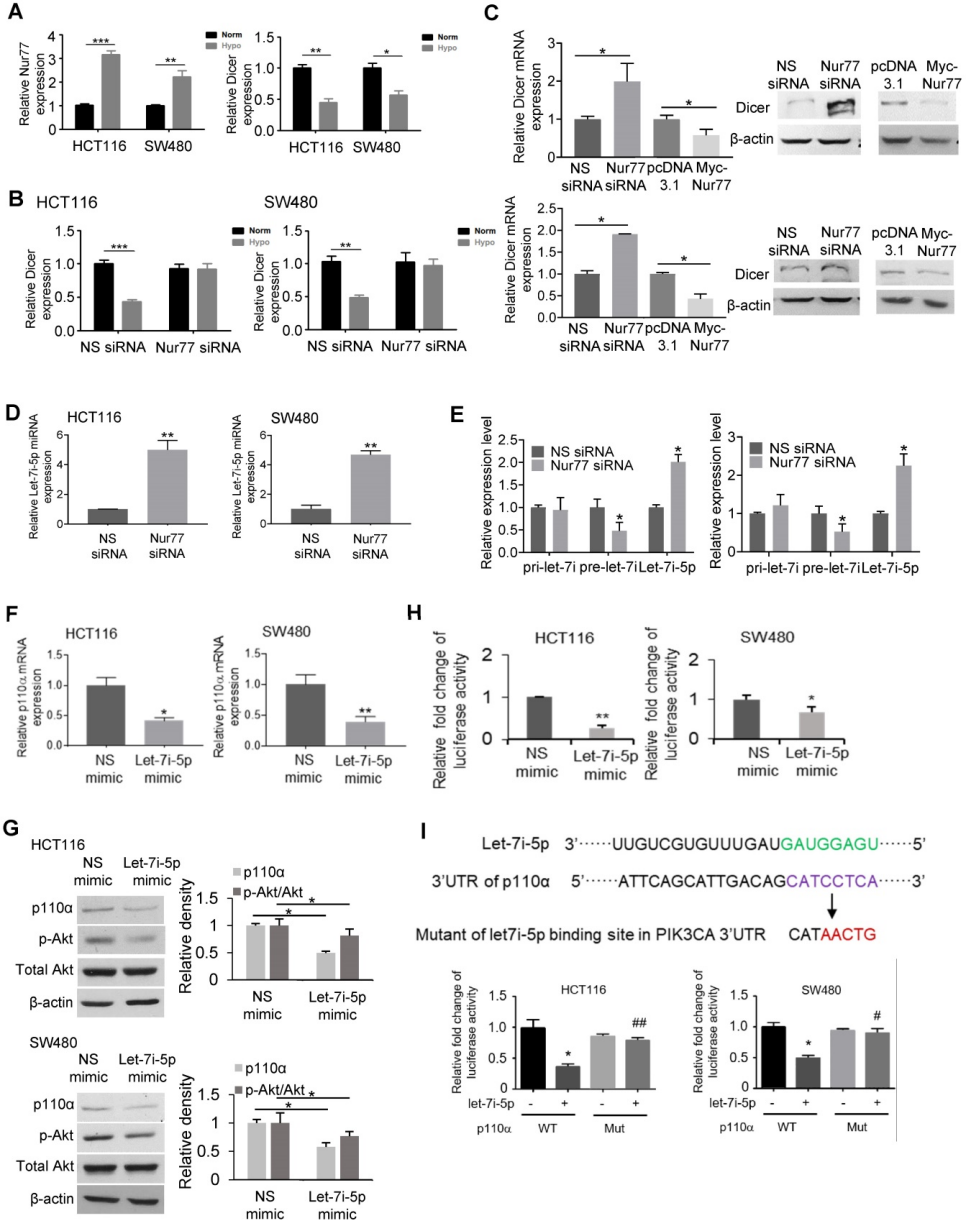
** Nur77 inhibits Dicer and let-7i-5p expression to induce p110α expression and Akt phosphorylation. (A)** HCT116 and SW480 cells were incubated at normoxia and hypoxia (1% O_2_) for 4 h. Expression of Nur77, Dicer, and GAPDH was determined by qPCR. **(B)** HCT116 and SW480 cells were transfected with non-specific (NS) or Nur77 siRNA, followed by incubation at normoxia or 1% O_2_. The mRNA expression of Dicer was determined by qPCR. GAPDH served as an internal control. **(C)** HCT116 or SW480 cells were transfected with NS siRNA, Nur77 siRNA, empty vector pcDNA3.1, or Nur77 construct as indicated, followed by 1% O_2_ incubation. The mRNA and protein expressions of Dicer were determined by qPCR and Western blotting. GAPDH and β-actin were included as an internal control.** (D)** CRC cells were transfected with NS or Nur77 siRNA, followed by 1% O_2_ incubation. Expression of let-7i-5p was determined by qPCR. U6 was included as an internal control. **(E)** CRC cells were transfected with the indicated siRNA or expression vectors, followed by hypoxic incubation. Levels of primary (pri-) let-7i, precursor (pre-)-let-7i and let-7i-5p were examined by qPCR. Pre-U6 and mature U6 were used as internal controls for pri-/pre-let-7i and let-7i-5p. **(F)** qPCR was performed to detect p110α mRNA levels, and **(G)** Western blotting was performed to detect p110α, p-Akt, and total Akt protein levels. β-actin was used as an internal control. **(H)** CRC cells were transfected with a luciferase reporter containing the putative p110α 3'UTR binding site in the presence of either NS or let-7i-5p mimic for 48 h. Luciferase activity of each sample was normalized with the activity of co-transfected Renilla luciferase. **(I)** The diagram shows the let-7i-5p putative binding site in the 3' UTR of p110α mRNA and its corresponding mutated sequence. SW480 and HCT116 cells were transfected with wild-type or mutated p110α 3'UTR luciferase reporter construct in the presence of either NS or let-7i-5p mimics for 48 h. Luciferase activity of each sample was normalized with the activity of co-transfected Renilla luciferase. For qPCR, relative quantification was achieved by normalization to the amount of GAPDH using 2^-ΔΔCt^ method. For Western blotting, the band intensities were quantified by densitometric analysis. Data is presented as mean ± SD of three biological replicates **(A)**, **(B)**, **(C)**,** (D)**,** (E)**,** (F)** for qPCR. Data is presented as mean ± SD., and one of three independent experiments is shown for Western blot **(C)**, **(G)**; and as mean ± SD for luciferase assay **(H)**, **(I)**. * or # indicates *P* < 0.05, ** or ## indicates *P* < 0.01, *** indicates *P* < 0.001, hypoxia compared with normoxia, Nur77 siRNA compared with NS siRNA in two-way ANOVA **(B)**; hypoxia compared with normoxia, Nur77 siRNA compared with NS siRNA, Myc-Nur77 compared with pcDNA3.1 and let-7i-5p mimic compared with NS mimic in paired Student's *t* test for the rest.

Numerous factors are known to regulate mRNA stability, including miRNAs, small non-coding RNAs that complementarily bind to the 3' untranslated regions (UTRs) of target mRNAs to regulate their degradation or translation 13. Dicer and Drosha are key enzymes that regulate miRNA biogenesis, and hypoxia has been shown to be a potent suppressor of Dicer and Drosha expression 14, 15. Therefore, we analyzed if Nur77 might interfere with Dicer and Drosha to disrupt miRNA production in the hypoxic CRC cells. Our results showed that hypoxia-mediated repression of Dicer transcription could be restored by Nur77 siRNA (Figure 4A-B). Overexpression of Nur77 strongly decreased Dicer protein level, while knocking down Nur77 enhanced Dicer expression (Figure 4C). The negative regulation of Dicer by Nur77 under hypoxia was not due to its direct binding to Dicer promoter as a Nur77 mutant lacking the DNA-binding domain (Nur77ΔDBD) could exert similar effect (Figure S4A). The impact of Nur77 on Dicer expression was specific as it had no effect on Drosha levels (Figure S4B).

The down-regulation of Dicer by Nur77 prompted us to identify miRNAs that could potentially regulate p110α. To this end, we used three prediction tools, namely TargetScan, RNAhybrid, and miRBase (miRanda) to predict potential regulators 16-18. Our analyses identified 15 miRNAs that potentially target p110α that were present at low levels in CRC. Among these miRNAs, we chose to study let-7i-5p since the low expression of let-7i was closely associated with distant metastasis and poor survival in CRC patients 19. qPCR analyses showed that let-7i-5p was significantly increased after Nur77 siRNA transfection (Figure 4D). Regulation of miRNA biogenesis could be controlled at the transcription and/or maturation of miRNAs. To better understand the role of Nur77 in miRNA processing, we examined the expression levels of let-7i-5p (the mature miRNA) and its two precursors, primary (pri)-let-7i and precursor (pre)-let-7i. Our results showed that under hypoxia, Nur77 siRNA significantly increased let-7i-5p, but decreased pre-let-7i (Figure 4E). The expression of pri-let-7i remained unaffected. Similar to full-length Nur77, Nur77ΔDBD also effectively decreased mature let-7i-5p levels (Figure S4C). These results collectively suggested that Nur77 did not interfere with pri-let-7i transcription or the cleavage of pri- into pre-let-7i, but affected the maturation of let-7i-5p from pre-let-7i. This was consistent with the specific suppressive role of Nur77 on Dicer, the enzyme responsible for cleaving pre-miRNA into mature miRNA.

We transfected HCT116 and SW480 cells with let-7i-5p mimic to validate if it would regulate p110α/Akt. Let-7i-5p mimic remarkably inhibited p110α expression and Akt phosphorylation compared to NS miRNA mimic (Figure 4F-G). We further constructed a luciferase reporter containing the putative let-7i-5p binding site present on the 3' untranslated region (UTR) of p110α. Transfection of the let-7i-5p mimic significantly repressed the luciferase activity compared to the control mimic (Figure 4H), confirming p110α suppression by let-7i-5p. To verify if let-7i-5p targets the predicted p110α binding site, HCT116 and SW480 cells were transfected with wildtype p110α 3'UTR luciferase reporter construct or its mutant lacking the putative let-7i-5p binding site in the presence of NS or let-7i-5p mimic. As shown in Figure 4I*,* p110α mutant-transfected cells lost let-7i-5p binding ability compared to wild-type, confirming a role for let-7i-5p in regulating p110α mRNA at the predicted binding site.

### Nur77 interacts with p63 to regulate Dicer expression

**Figure 5:**
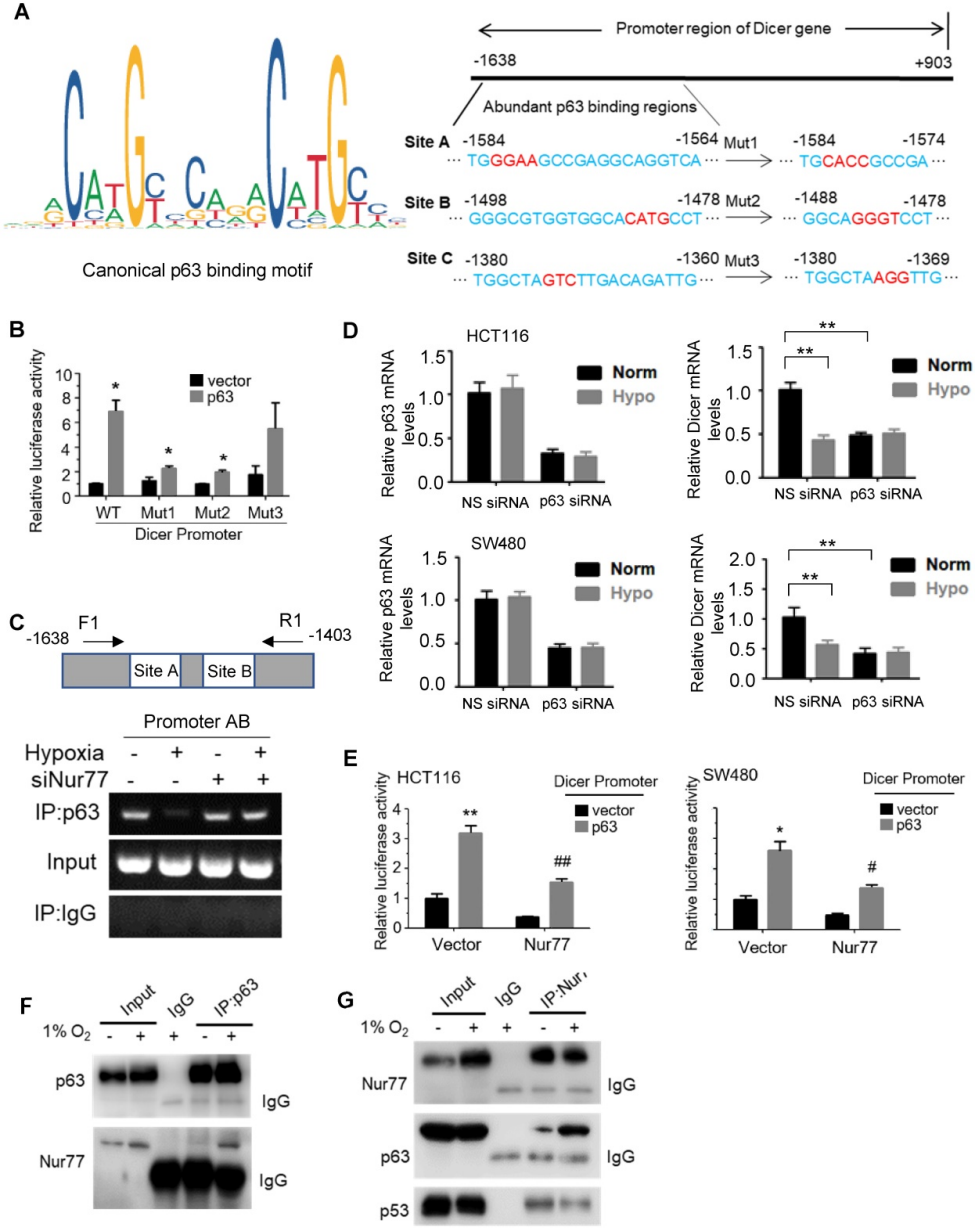
** Nur77 inhibits Dicer expression under hypoxia in a p63-dependent manner. (A)** Canonical p63 binding sequence analyzing with JASPAR (http://jaspar.genereg.net/) (left panel). The sequences and positions of putative p63 response elements in the Dicer promoter are shown. Three mutants, Mut1, Mut2, and Mut3, were constructed for the subsequent luciferase assays (right panel). **(B)** HEK293T cells were co-transfected with the pGL3-Dicer-prom plasmid (WT) or its mutants, Renilla luciferase constructs, and p63-expressing plasmid for 24 h, followed by measurements of luciferase activities. **(C)** HCT116 cells were transfected with Nur77 siRNA or non-specific (NS) siRNA for 48 h under normoxia or hypoxia. The cell lysates were incubated with anti-p63 antibody, and IgG was used as a negative control. Eluted DNA from the immunoprecipitates was amplified by PCR using primers covering Site A and Site B (Promoter AB). **(D)** HCT116 and SW480 cells were transfected with NS siRNA or p63 siRNA for 48 h, followed by incubation at 1% O_2_ for 4 h. Expressions of p63, Dicer, and GAPDH were determined by qPCR. **(E)** HCT116 and SW480 cells were co-transfected with the pGL3-Dicer-promoter plasmid, Renilla luciferase constructs, and the indicated plasmids (Empty vector, Nur77 or p63) for 24 h. Renilla luciferase activity was used to normalize for transfection efficiency. **(F)** HCT116 cells incubated at normoxia or 1% O_2_ for 24 h were lysed and immunoprecipitated with p63 antibody. Immunoblotting was performed to detect p63 and Nur77. **(G)** Whole-cell lysates of HCT116 cells incubated at normoxia or 1% O_2_ for 24 h were immunoprecipitated with Nur77 antibody. Immunoblotting was performed with antibodies against Nur77, p63, and p53. For qPCR, relative quantification was achieved by normalization to the amount of GAPDH using 2^-ΔΔCt^ method. Data is presented as mean ± SD., and one of three independent experiments is shown **(B)**, **(C)**, **(E)**, **(F)**, **(G)**, or as mean ± SD. of three biological replicates **(D)**. * or # indicates *P* < 0.05, ** or ## indicates *P* < 0.01, Muts compared with wild type, p63 compared with empty vector, Nur77 or p63 compared with empty vector in paired Student's *t* test **(B)**,** (E)**; Hypoxia compared with normoxia, p63 siRNA compared with NS siRNA in two-way ANOVA** (D)**.

We next explored the underlying mechanisms by which Nur77 regulates Dicer expression. In the Dicer promoter, any putative Nur77 response elements could not be detected, raising the possibility that Nur77 did not bind directly to the Dicer promoter to modulate its activity. Dicer has been shown to be a p53-inducible gene 20, while Nur77 was reported to interact with and suppress p53 transcriptional activity 21. However, we found that Nur77 could regulate Dicer expression in both HCT116 (with wild-type p53) and SW480 (with mutant p53) cell lines, suggesting that its activity could be p53-independent. Therefore, we asked if p63, a homolog of p53, might be involved in regulating Dicer. The canonical p63 binding sequence has been illustrated in Figure 5A. Using the online JASPAR database (http://jaspar.genereg.net/), we found three putative p63-binding sites (A, B, and C) located between -1638 and -1380 bp in the Dicer promoter region (Figure 5A). We thus cloned this region and introduced mutations into each individual site. Reporter assays showed that p63 transfection could potently activate the Dicer promoter (Figure 5B). Such activation could be retained in Mut3 (site C mutant) but lost in Mut1 (site A mutant) and Mut2 (site B mutant), suggesting that p63 may bind to sites A and B. Using primers covering sites A and B (Promoter AB), our ChIP assays showed that p63 could bind to this region under normoxia, while hypoxia impeded the binding of p63 to the Dicer promoter (Figure 5C), suggesting that it is an *in situ* binding region for p63 in the CRC cells. In contrast, p63 could not bind the control region covering site C (Promoter C) (Figure S5A). ChIP and reporter assays showed that silencing of Nur77 could reverse the hypoxia-induced impairment on p63 binding to the Dicer promoter (Figure 5C and Figure S5B). The critical role of p63 in activating Dicer transcription was further demonstrated. Dicer mRNA was expressed in CRC cells under normoxia, but inhibited upon silencing p63, suggesting that p63 is essential for Dicer transcription. Suppression of Dicer transcription was consistently seen under hypoxic stress or in combination of p63 siRNA (Figure 5D). The p63-dependent Dicer promoter activity was abrogated upon co-transfection of Nur77 or Nur77∆DBD (Figure 5E, Figure S5C). To explore the underlying mechanism, we asked if Nur77 could interact with p63. Indeed, we found that Nur77 and p63 co-precipitated together, either endogenously or in ectopic transfection, both of which could be enhanced by hypoxia (Figure 5F-G and Figure S5D). Hypoxia could also induce Nur77∆DBD interaction with p63 (Figure S5D). Interestingly, we also found that while Nur77 could interact with p53, there was no difference between normoxic and hypoxic conditions (Figure 5G). We thus demonstrate that Nur77 acts as a specific negative regulator of Dicer transcription through its direct interaction with p63 under hypoxic condition.

### Inhibition of Dicer/ let-7i-5p by Nur77 induces CSC phenotype

**Figure 6:**
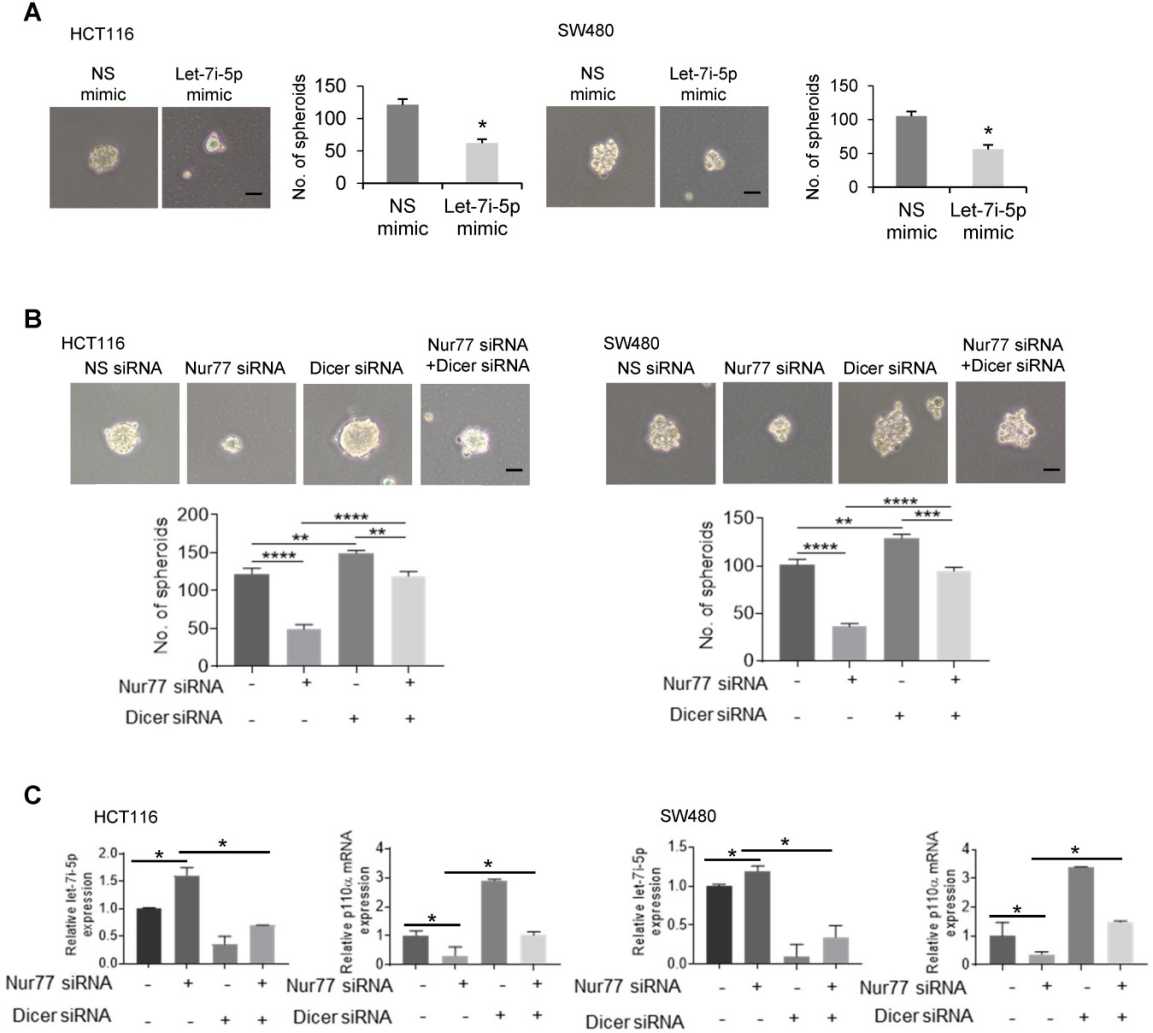
** Inhibition of Dicer/ let-7i-5p by Nur77 induces CSC phenotypes.** HCT116 or SW480 cells were transfected with **(A)** NS or let-7i-5p mimic, or with **(B)** non-specific (NS), Nur77, or Dicer siRNA, as indicated for 48 h, followed by 1% O_2_ incubation. Cells were cultured as tumor spheres. Representative spheres were photographed, and the numbers of spheres were recorded from five random fields of view. Bar, 50 µm. **(C)** Total RNA was extracted and analyzed by qPCR to determine the expression levels of let-7i-5p and p110α. GAPDH and U6 were included as internal controls. For qPCR, relative quantification was achieved by normalization to the amount of GAPDH using 2^-ΔΔCt^ method. Data is presented as mean ± SD., and one of three independent experiments is shown **(A)**, **(B)**, or as mean ± SD. of three biological replicates **(C)**. * indicates *P* < 0.05, ** indicates *P* < 0.01, *** indicates *P* < 0.001, **** indicates *P* < 0.0001, let-7i-5p mimic compared with NS mimic in paired Student's *t* test **(A)**. Nur77 siRNA compared with NS siRNA, Dicer siRNA compared with NS siRNA, and Nur77 siRNA together with Dicer siRNA compared with Nur77 siRNA or Dicer siRNA only in two-way ANOVA **(B)**, **(C)**.

Given that hypoxia-induced Nur77 could enrich CSC properties (Figure 1), we asked if Dicer and let-7i-5p were also involved in inducing the CSC phenotype. To this end, a let-7i-5p mimic was used, whose expression was confirmed by qPCR (Figure S6A). We showed that let-7i-5p mimic could markedly suppress hypoxia-induced sphere-forming abilities by reducing both the number and size of CRC spheres (Figure 6A). The specific inhibition of tumor sphere formation and p110α expression by Nur77 siRNA could be rescued by let-7i-5p inhibitor (Figure S6B-S6C). Nur77 depletion could not inhibit tumor sphere formation when Dicer was co-depleted (Figure 6B). In addition, qPCR demonstrated that Nur77 siRNA failed to up-regulate let-7i-5p or down-regulate p110α/p-Akt in the presence of Dicer siRNA (Figure 6C). These results demonstrated that Nur77 repressed let-7i-5p *via* Dicer to prevent p110α degradation and casually induced the CSC phenotype.

### Nur77 enhances HCT116 xenograft growth and metastasis *in vivo via* the Dicer-let-7i-5p-p110α-AKT-mTOR signaling axis

**Figure 7:**
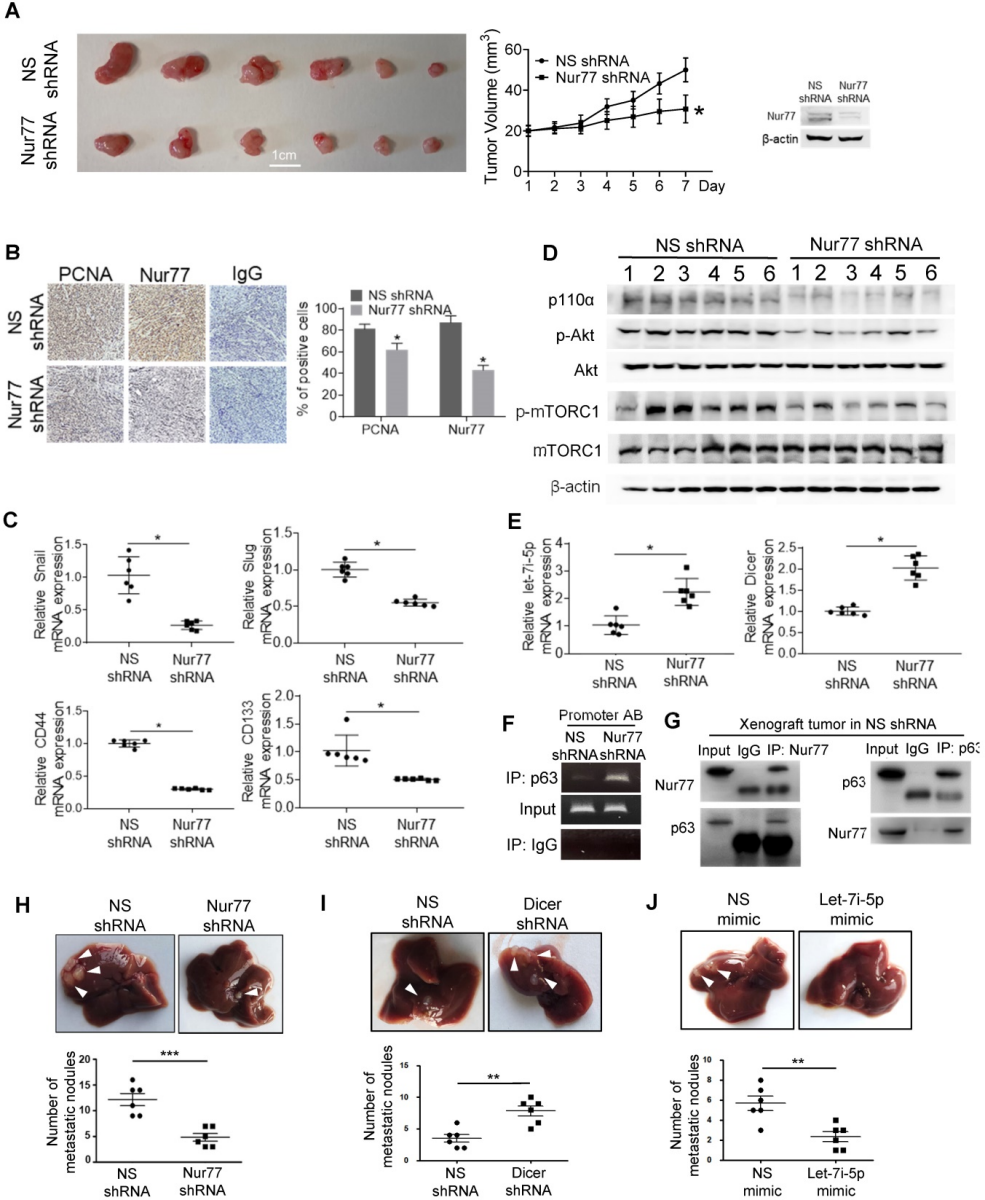
** Nur77 enhances HCT116 xenograft growth and metastasis *in vivo via* the p110α/AKT/mTOR pathway. (A)** BALB/c nude mice were randomly allocated to two groups and injected subcutaneously with either Nur77 shRNA or non-specific (NS) shRNA stable transfected HCT116 cells (2×10^6^) (n = 6, repeated three times). Tumor volumes were recorded and measured daily. Mice were euthanized 7 days later and tumors were removed and photographed. Nur77 knockdown was confirmed with Western blot.** (B)** Nur77 and PCNA expression levels were detected by immunohistochemistry in the subcutaneous tumor tissues (200×). Percentages of PCNA- and Nur77-positive cells were analyzed in Nur77 shRNA and NS shRNA.** (C)** The expression of Snail, Slug, CD44 and CD133 was determined by qPCR. GAPDH was used as internal control. **(D)** The protein levels of p110α, p-Akt, total Akt, p-mTOR, total mTOR and β-actin were examined by Western blotting.** (E)** The expression of Dicer and let-7i-5p was determined by qPCR. GAPDH and U6 were used as internal controls. (**F**) ChIP assay was performed to detect the binding levels of p63 on Dicer promoter in xenograft tumor samples in Nur77 shRNA or NS shRNA groups. The tumor lysates were incubated with anti-p63 antibody, and IgG was used as a negative control. Eluted DNA from the immunoprecipitates was amplified by PCR using primers covering Site A and Site B (Promoter AB). **(G)** The interactions between Nur77 and p63 protein in xenograft tumor samples were determined by Co-IP assay. Tumor lysates from NS shRNA group were immunoprecipitated with Nur77 or p63 antibody, then detected by immunoblotting.** (H)(I)(J)** HCT116 (5×10^5^) cells stably expressing NS shRNA or Nur77 shRNA, NS shRNA or Dicer shRNA, NS mimic or let-7i-5p mimic were intra-splenically injected into mice to establish the metastatic liver model (n = 6, repeated three times). Mice were euthanized 6 weeks later and livers were removed and photographed. Representative images of metastatic liver nodules are shown and the numbers of metastatic nodules were statistically analyzed. For tumor volumes comparison, data is presented as mean ± SEM of 6 samples from different mice, and the photograph of all tumors on the 7^th^ day is shown **(A)**. * indicates *P* < 0.05 in paired Student's *t* test (Nur77 shRNA compared with NS shRNA). DAB positive signal density was quantified using Image J, the data is presented as mean ± SEM **(B)**, * indicates *P* < 0.05 in paired Student's *t* test (Nur77 shRNA compared with NS shRNA). For qPCR, relative quantification was achieved by normalization to the amount of GAPDH using 2^-ΔΔCt^ method. Data is presented as individual values and mean ± SEM of 6 samples from different mice **(C)**, **(E)**, **(H), (I), (J)**. * indicates *P* < 0.05, ** indicates *P* < 0.01, *** indicates *P* < 0.001 in paired Student's *t* test (Nur77 or Dicer shRNA compared with NS shRNA, and let-7i-5p mimic compared with NS mimic).

To verify Nur77 function and signaling *in vivo,* HCT116 cells were stably transfected with Nur77 shRNA. The depletion efficiency of Nur77 was shown (Figure 7A and B). As expected, Nur77 shRNA strongly inhibited subcutaneous tumor formation (Figure 7A) and reduced PCNA expression, a cell proliferative marker, in xenograft tumors (Figure 7B). Consistent with the *in vitro* data, Nur77 shRNA tumors revealed significantly increased expression of Dicer and Let-7i-5p compared to NS shRNA controls, which coincides with its inhibition of mesenchymal (snail and slug) and CSC (CD44 and CD143) markers (Figure 7C). Nur77-benefited EMT was further evidenced that Nur77 shRNA-enhanced Dicer expression, up-regulation of E-cadherin and down-regulation of N-cadherin, snail and slug at the protein levels (Figure S7A). Consistently, Nur77 shRNA significantly reduced p110α protein and Akt phosphorylation levels, as well as inhibition of mTORC1 activity (Figure 7D). We further confirmed that Nur77 shRNA greatly promoted the capability of p63 to bind to Dicer promoter (Figure 7F) and that the endogenous interaction of Nur77 with p63 was reproducible in *in vivo* (Fig. 7G). Importantly, we showed that the liver metastasis of CRC cells was inhibited by Nur77 shRNA and let-7i-5p mimic (Figure 7H and 7J), but was promoted by Dicer shRNA (Figure 7I). Together with our *in vitro* observations, our findings thus demonstrate that Dicer, Let-7i-5p and its downstream p110α/Akt/mTORC1 play a critical role in Nur77-driven CRC growth and metastasis.

### Nur77/Dicer/p110α gene expression associates with patient survival

**Figure 8:**
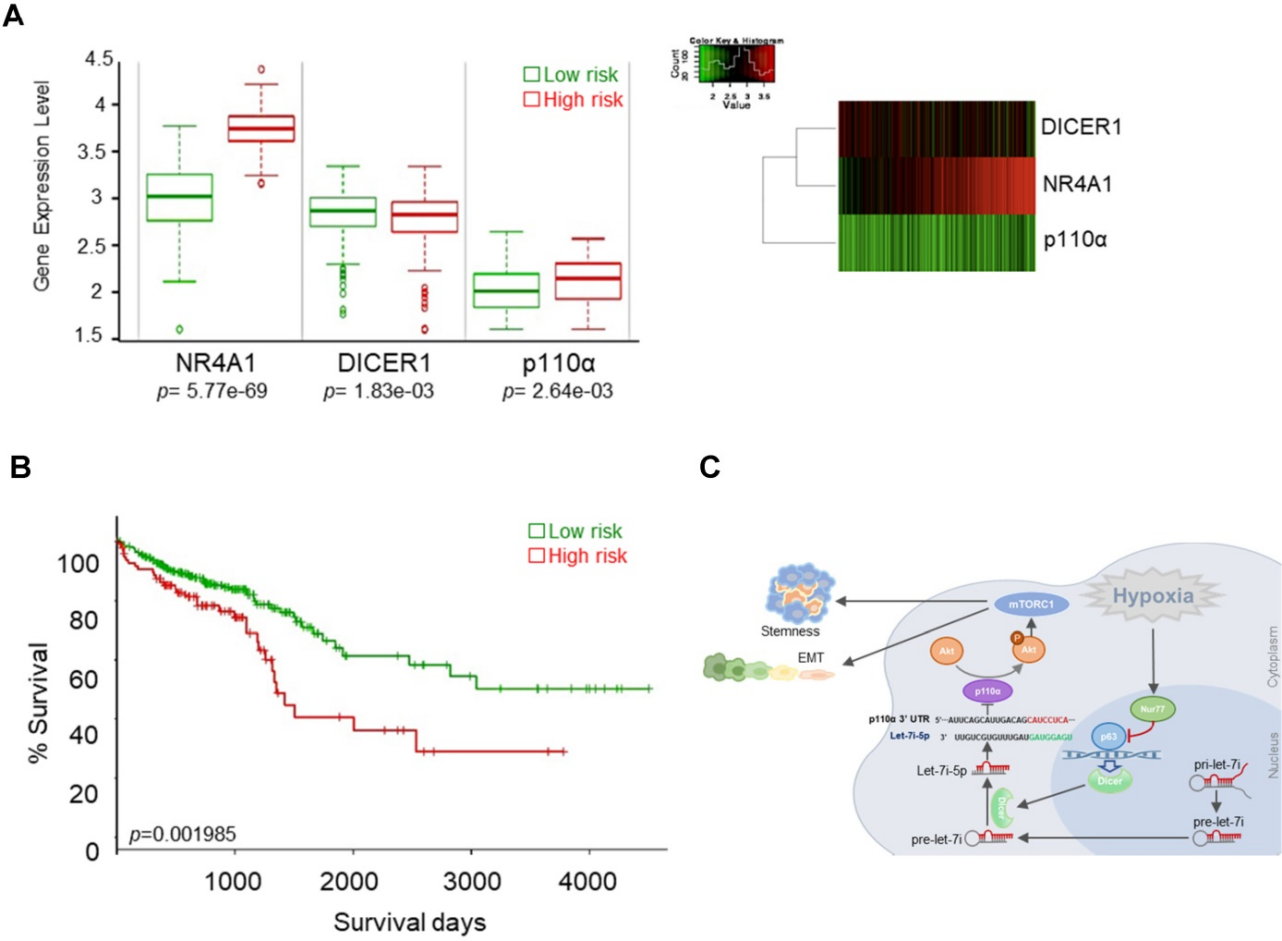
** Nur77/Dicer/p110α gene expression associates with patient survival. (A)** Using the web-based tool SurvExpress, patients from the TCGA colon adenocarcinoma (COAD) database were split into low-risk (green) and high-risk (red) groups after ranking the samples by their prognostic indexes. Box plot (left) and heat-map (right) visualizing the gene expression values for NR4A1, DICER1, and p110α are shown.** (B)** Kaplan-Meier survival curves of the low- and high-risk groups were generated. *P*-values correspond to the log-rank test and t-test for the Kaplan-Meier curves and box plot, respectively. **(C)** Summary of the proposed signaling mediated by hypoxia-induced Nur77.

We evaluated the possible clinical relevance of the Nur77/Dicer/p110α axis by analyzing the TCGA CRC unstratified (pooled) dataset using SurvExpress, an online tool for survival analysis and risk assessment of cancer datasets 22, 23. Patients were split into a low- and a high-risk group after ranking the samples by their prognostic indexes. Patient samples belonging to the high-risk group were found to express higher Nur77 (NR4A1), lower Dicer (DICER1), and higher p110α (PIK3CA) (Figure 8A). Kaplan-Meier plot showed that this group of patients had shorter survival than patients in the low-risk group (Figure 8B). Figure 8C summarizes the signaling pathway described in this study.

## Discussion

Hypoxia is one of the main obstacles in treating solid tumors. Tumors growing under hypoxia exhibit an aggressive phenotype, resulting in increased metastasis and enhanced resistance to conventional radiotherapy and chemotherapy 24. Since tumor hypoxia is prevalent across cancer types, it is imperative to understand at the molecular level how cancer cells respond to hypoxia. Nur77 overexpression has been observed in many solid tumors, such as CRC, lung, and breast cancers 25-27. The expression of Nur77 could be hyper-stimulated by hypoxic conditions and the dysregulation of Nur77 was shown to promote metastasis 7. Furthermore, Nur77 could transcriptionally activate the expression of β1-integrin, β4-integrin, and Nanog, involved in cancer cell invasion and stemness 28, 29. This study has provided evidence that Nur77 could enhance both EMT and CSC properties of CRC cells under hypoxic condition and uncovered a novel non-genomic route for mediating these effects.

PI3K is a crucial heterodimeric lipid kinase in cancer development with three classes comprising different structures and biochemical features. Among them, Class IA is the most common type consisting of a regulatory (p85) and a catalytic subunit (p110) 9. Activating mutations of the p110α subunit, encoded by the gene PIK3CA, are found in 20-30% of CRC patients 30. However, high p110α expression did not entirely correlate with p110α mutation, implying alternative mechanisms responsible for p110α overexpression 31. Our results in the current study identified hypoxia-induced Nur77 as one of the causative factors.

miRNAs are small non-coding regulatory RNAs that post-transcriptionally control gene expression and are vital regulators of many cellular processes and could serve as potential targets or biomarkers in the clinic 32-34. Although Nur77 was regulated by various miRNAs, including miR-124, miR-15a, and miR-224 35, we showed here, for the first time, that Nur77 could also suppress miRNA biogenesis *via* Dicer, which cleaves pre-miRNA into mature miRNA. Low expression of Dicer, frequently observed in various malignancies, was reported to be associated with poor clinical outcome 36, 37. In CRC, Dicer impairment was shown to induce tumor initiation and metastasis 38. Here we identified a suppressive role of Nur77 on Dicer and its downstream let-7i-5p miRNA during hypoxia, providing an unprecedented link between Nur77 and post-transcriptional regulation of other genes through miRNAs.

In humans, the let-7 miRNA family consists of 10 mature members. As one of the earliest tumor suppressor miRNAs discovered, let-7 was shown to be under-expressed in many human malignancies 39. Although let-7i is relatively less studied than other family members, such as let-7a/b/c, it is highly relevant to CRC as it has been strongly associated with both distant metastases and poor patient survival 19. The regulation of p110α/p-Akt by let-7i reported here has identified a new tumor-suppressive role for this miRNA during CRC progression.

We found that Nur77 could regulate Dicer expression independent of its transactivation ability. This is not unexpected since Nur77 has been shown to be a nucleocytoplasmic shuttling protein with various genomic and non-genomic roles during cancer progression 40. Our observation of a p53-independent Dicer regulation identified p63, a p53 homolog, as a novel interacting partner of Nur77. TheNur77-p63 interaction strongly inhibited p63-dependent transcription of Dicer under hypoxia, resulting in reduced biogenesis of the tumor-suppressive let-7i-5p. These findings revealed a new pathway underlying the p53-independent actions of Nur77 among various cancers and other disease types.

## Conclusions

To summarize, our data demonstrated a novel non-genomic mechanism of Nur77 to enhance cancer EMT and stemness properties. The inhibition of Dicer/let-7i and subsequent activation of the oncogenic p110α/Akt pathway suggest an attractive therapeutic potential for Nur77.

## Supplementary Material

Supplementary figures and tables.
