# Supplementary material for: Hypoxia-induced Nur77 activates PI3K/Akt signaling via suppression of Dicer/let-7i-5p to induce epithelial-to-mesenchymal transition
Source: Theranostics. 2021 Jan 19;11(7):3376–91. doi: 10.7150/thno.52190 (PMC7847671; doi:10.7150/thno.52190)
Supplement: Supplementary file 1 — Supplementary figures and tables. [file thnov11p3376s1.pdf]

Figure S1. Nur77 mediates hypoxia-induced CSC-phenotypes *in vitro* but no obvious changes were found under normoxia. (A) HCT116 and SW480 cells were transfected with non-specific (NS) or Nur77 sRNA for 48 h; empty vector pcDNA3.1 or Nur77 constructs for 24 h followed by incubation under normoxia. The cells were fixed and observed for EMT morphologic changes. Representative scattered colonies were imaged and scattered colonies were scored from five random fields of view. Expression of EMT markers Snail, Slug, E-cadherin, and N-cadherin were determined by qPCR. **(B)** Transfected cells were allowed to form tumor spheres followed by incubation under normoxia. Spheres were photographed. Expression of cancer stem cell markers CD44, CD133, EpCAM, and ALDH-1 were detected by qPCR. GAPDH served as an internal control. Relative quantification was achieved by normalization to the amount of GAPDH using $2^{-\Delta\Delta Ct}$ method. **(C)** HCT116 and SW480 cells were transfected with NS siRNA or Nur77 siRNA for 48 h; empty vector pcDNA3.1 or Nur77 constructs for 24 h followed by incubation at 1% O_2 and subjected to the serial spheroid formation assay. Spheres were photographed and counted. Data is presented as mean ± SD., and one of three independent experiments is shown **(A)**, **(B)**, **(C)**, or as mean ± SD. of three biological replicates **(A)**; * indicates $P < 0.05$ in paired Student's *t* test (Nur77 siRNA compared with NS siRNA and Myc-Nur77 compared with pcDNA3.1). Bar, 50 μm.

Figure S1

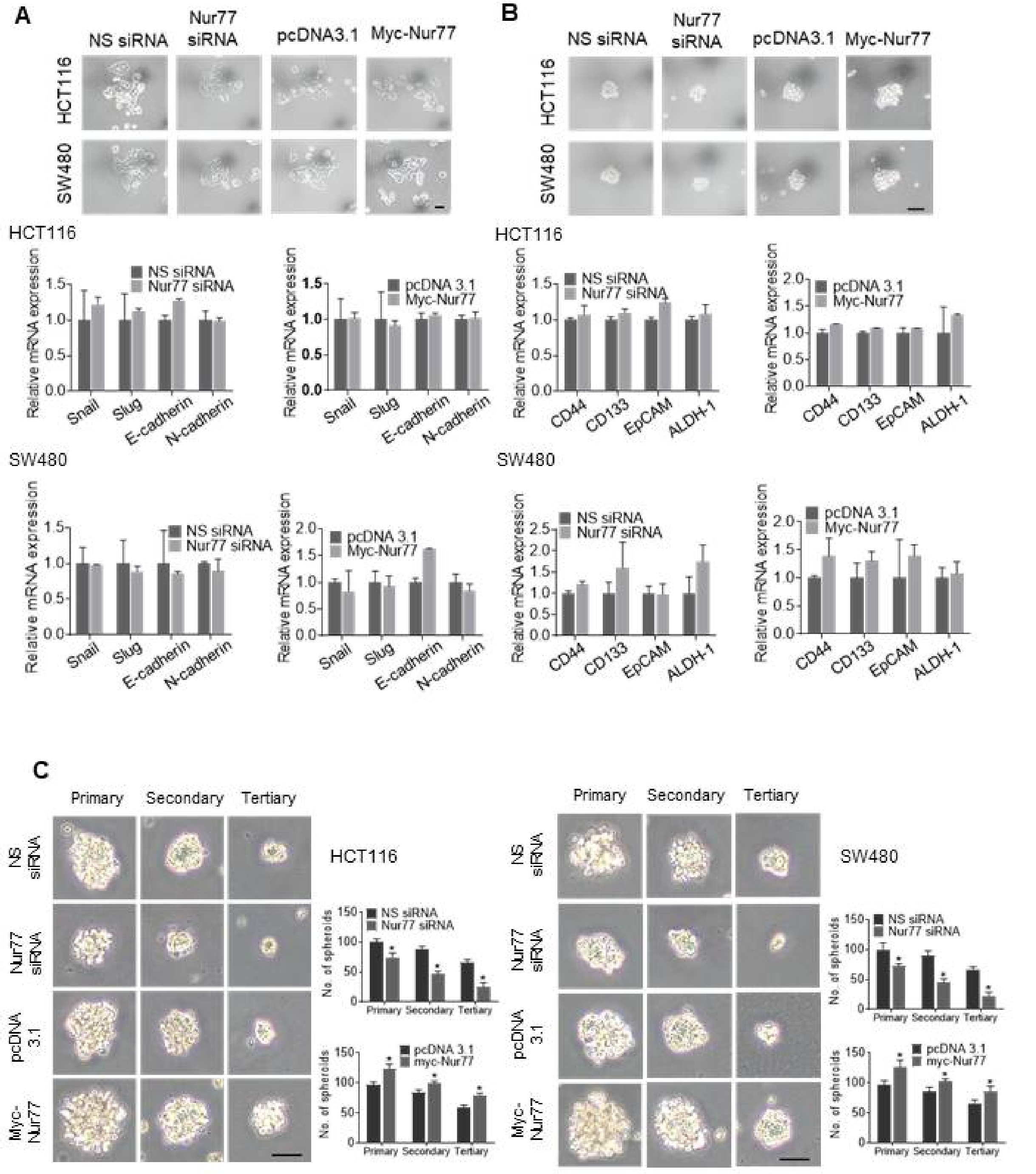

A
NS siRNA
Nur77 siRNA
pcDNA3.1
Myc-Nur77
HCT116
SW480
B
NS siRNA
Nur77 siRNA
pcDNA3.1
Myc-Nur77
HCT116
SW480
HCT116
Relative mRNA expression
NS siRNA
Nur77 siRNA
pcDNA 3.1
Myc-Nur77
Snail
Slug
E-cadherin
N-cadherin
CD44
CD133
EpCAM
ALDH-1
SW480
C
Primary
Secondary
Tertiary
NS siRNA
Nur77 siRNA
pcDNA 3.1
Myc-Nur77
HCT116
SW480
No. of spheroids
myc-Nur77

Figure S2. No significant effects were observed under normoxia.

HCT116 and SW480 cells were transfected with non-specific (NS) or Nur77 siRNA for 48 h; empty vector pcDNA3.1 or Nur77 constructs for 24 h followed by incubation under normoxia. Spheres were collected for western blot. **(A)** Expression of p110α, p-Akt, total Akt were detected by Western blot. **(B)** Expression of p110α mRNA was detected by qPCR. β-actin served as an internal control for Western blots and GAPDH for qPCR. Relative quantification was achieved by normalization to the amount of GAPDH using $2^{-\Delta\Delta Ct}$ method. Data is presented as one of three independent experiments **(A)**, or as mean ± SD. of three biological replicates **(B).**

Figure S2

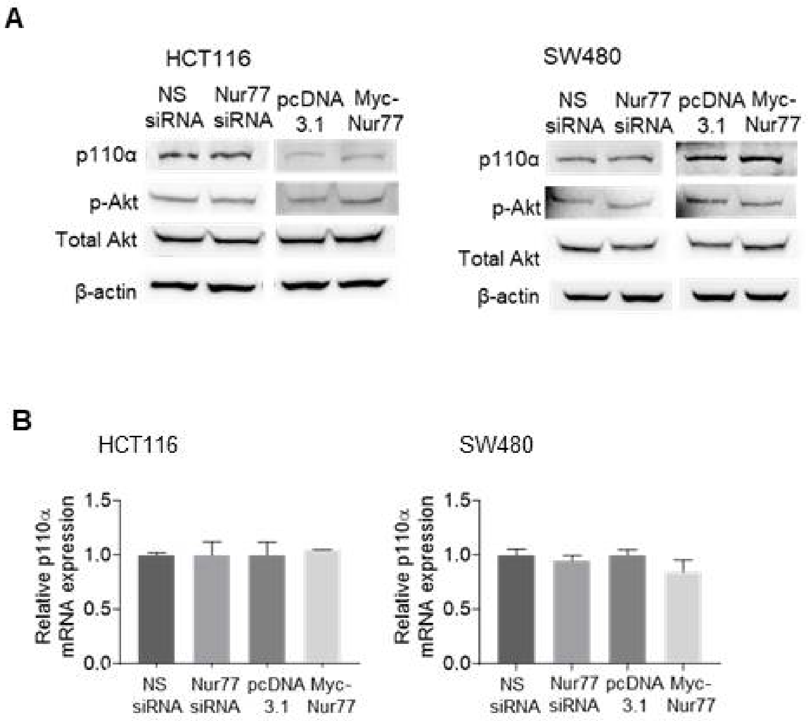
A
HCT116
NS siRNA
Nur77 siRNA
pcDNA 3.1
Myc-Nur77
p110α
p-Akt
Total Akt
β-actin
SW480
NS siRNA
Nur77 siRNA
pcDNA 3.1
Myc-Nur77
p110α
p-Akt
Total Akt
β-actin
B
HCT116
SW480
Relative p110α mRNA expression
1.5
1.0
0.5
0.0
NS siRNA
Nur77 siRNA
pcDNA 3.1
Myc-Nur77

Figure S3. Nur77 fails to enhance p110α mRNA stability in HCT116 and in SW480 cells under normoxia. HCT116 cells or SW480 cells transfected with non-specific (NS) or Nur77 siRNA were treated with actinomycin D (Act-D; 5 μg/ml) for different time periods (0, 2, 4, 8, and 24 h). Total RNA was harvested to determine p110α expression level. GAPDH was included as an internal control. Relative quantification was achieved by normalization to the amount of GAPDH using $2^{-\Delta\Delta Ct}$ method. Data is presented as mean ± SD. of three biological replicates**.**

Figure S3

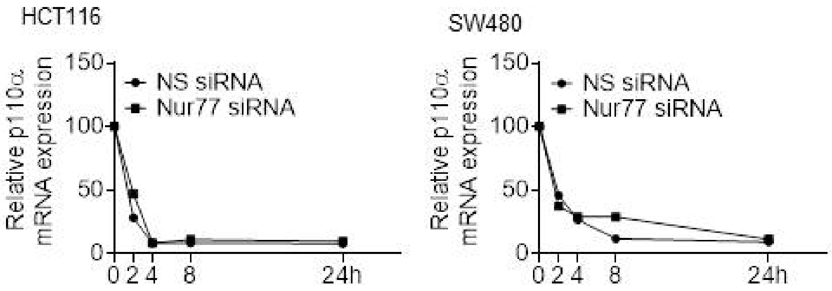
HCT116
Relative p110α mRNA expression
150
100
50
0
NS siRNA
Nur77 siRNA
0 2 4 8 24h
SW480
Relative p110α mRNA expression
150
100
50
0
NS siRNA
Nur77 siRNA
0 2 4 8 24h

Figure S4. Nur77 does not affect the expression levels of Drosha, but Nur77 ΔDBD inhibits Dicer expression and modulates let-7i-5p biogenesis. (A) HCT116 or SW480 cells were transfected with pcDNA3.1, or Nur77ΔDBD construct as indicated, followed by 1% O_2 incubation. The mRNA and protein expressions of Dicer were determined by qPCR and Western blotting. GAPDH and β-actin were included as an internal control. **(B)** HCT116 or SW480 cells were treated with non-specific (NS) siRNA, Nur77 siRNA, empty vector pcDNA3.1 or Nur77 constructs, followed by incubation at hypoxia. The mRNA and protein expression of Drosha were determined by qPCR and Western blotting. GAPDH and β-actin were included as an internal control. **(C)** HCT116 and SW480 cells were transfected with the Nur77ΔDBD expression vectors, followed by nomorxic incubation. Levels of primary (pri-) let-7i, precursor (pre-)-let-7i and let-7i-5p were examined by qPCR. Pre-U6 and mature U6 were used as internal controls for pri-/pre-let-7i and let-7i-5p. For qPCR, relative quantification was achieved by normalization to the amount of GAPDH using $2^{-\Delta\Delta Ct}$ method. Data is presented as mean ± SD., and one of three independent experiments is shown **(A)**, **(B)**, or as mean ± SD. of three biological replicates **(A)**, **(B)**, **(C)**; * indicates $P < 0.05$, ** indicates $P < 0.01$, *** indicates $P < 0.001$ in paired Student's *t* test. (Nur77 siRNA compared with NS siRNA and Myc-Nur77 or Nur77 ΔDBD compared with pcDNA3.1.)

Figure S4

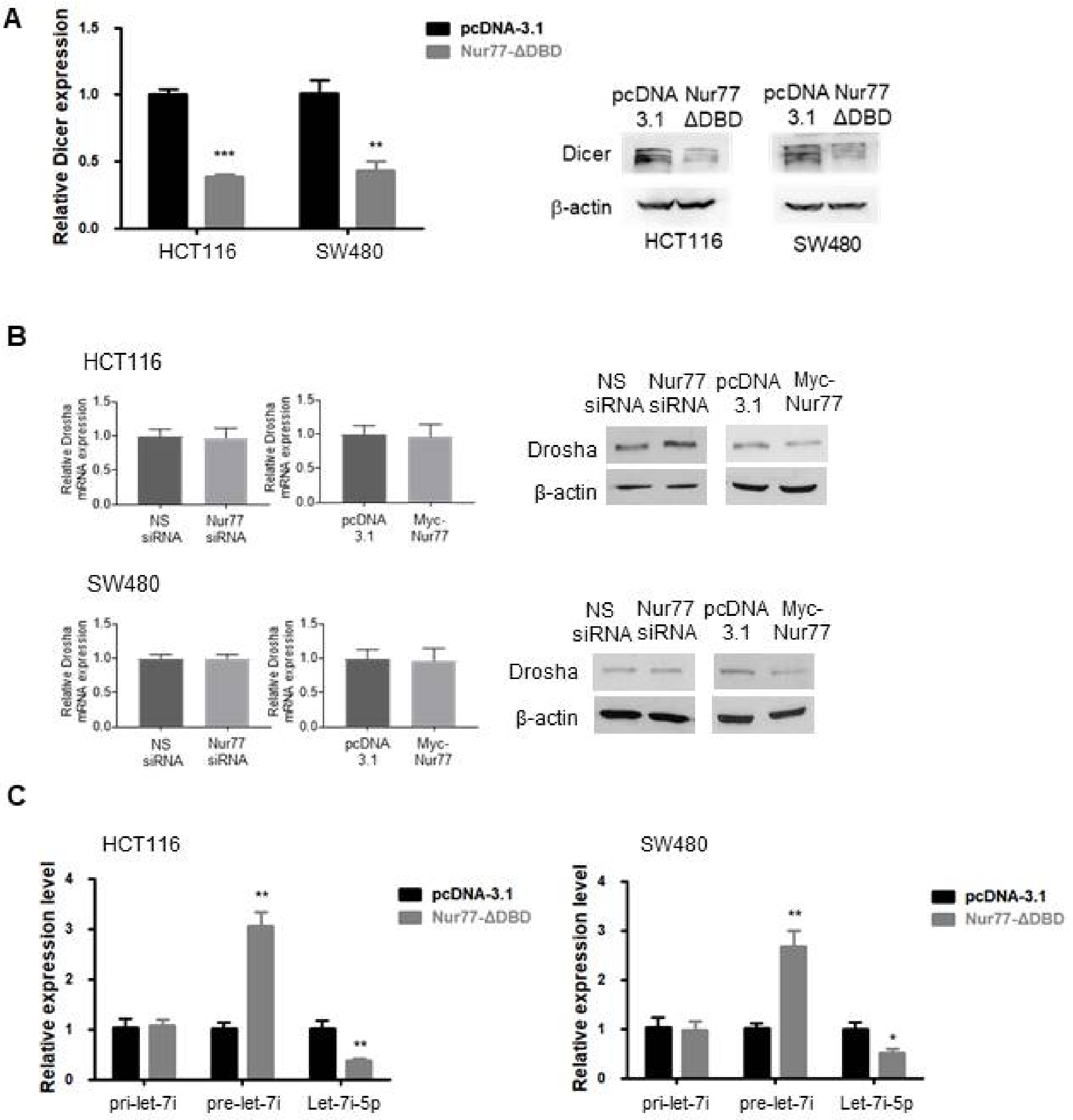
A
Relative Dicer expression
1.5
1.0
0.5
0.0
***
**
HCT116
SW480
pcDNA-3.1
Nur77-ΔDBD
pcDNA 3.1
Nur77 ΔDBD
Dicer
β-actin
B
HCT116
SW480
Relative Drosha mRNA expression
NS siRNA
Nur77 siRNA
pcDNA 3.1
Myc-Nur77
Drosha
C
Relative expression level
pri-let-7i
pre-let-7i
Let-7i-5p
*

Figure S5. Nur77ΔDBD represents the interaction ability of Nur77 with p63. (**A**) siRNA HCT116 cells were transfected with Nur77 siRNA or non-specific (NS) siRNA for 48 h under normoxia or hypoxia, respectively. The cell lysates were incubated with anti-p63 antibody, and IgG was used as a negative control. Eluted DNA from the immunoprecipitates was amplified by PCR using primers covering Site C (Promoter C). **(B)** HCT116 and SW480 cells were co-transfected with the Dicer promoter construct companied with Nur77 siRNA or NS siRNA for 48 h under hypoxia, detected by luciferase reporter assays. **(C)** HCT116 and SW480 cells were co-transfected with the pGL3 Dicer promoter plasmid, Renilla luciferase constructs, and the indicated plasmids (Empty vector, Nur77ΔDBD or p63) for 24 h. Renilla luciferase activity was used to normalize for transfection efficiency. **(D)** HEK293T cells were transfected GFP-p63 expressing plasmids with Myc vector, Myc-Nur77 or Myc-Nur77ΔDBD under normoxia or hypoxia, separately. Whole-cell lysates were subjected to IP with anti-Myc beads. Immunoblotting was performed with anti-Myc and anti-GFP antibodies. Data is presented as mean ± SD., and one of three independent experiments is shown **(A)**, **(D)**; * or # indicates $P < 0.05$, ** or ## indicates $P < 0.01$ in paired Student's t test (Nur77 siRNA compared with NS and Nur77 ΔDBD compared with empty vector).

Figure S5

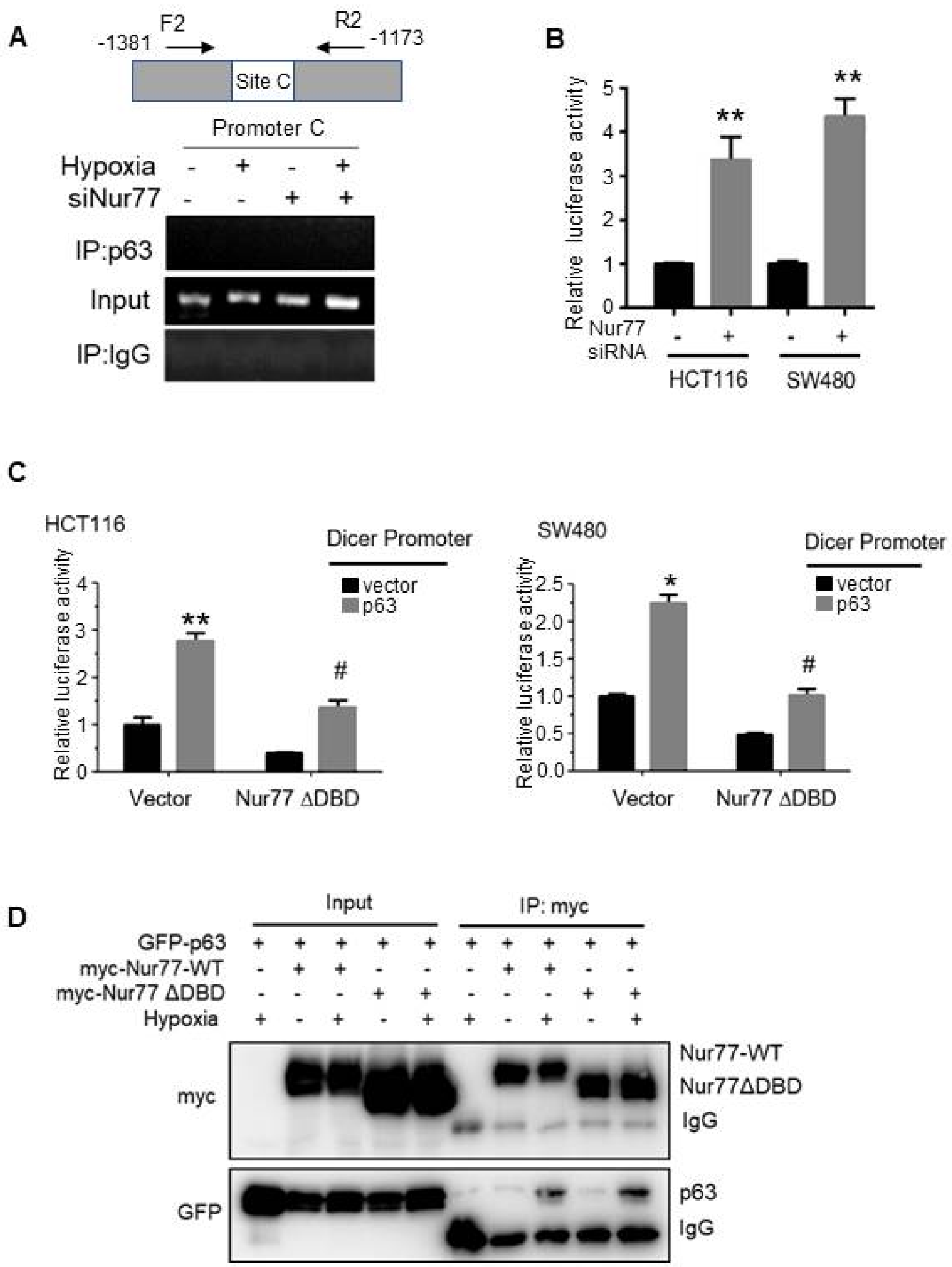

A
F2
R2
-1381
-1173
Site C
Promoter C
Hypoxia - + - +
siNur77 - - + +
IP:p63
Input
IP:IgG
B
Relative luciferase activity
5
4
3
2
1
0
**
**
Nur77 siRNA - + - +
HCT116
SW480
C
HCT116
Dicer Promoter
vector
p63
Relative luciferase activity
4
3
2
1
0
**
#
Vector
Nur77 ΔDBD
SW480
Dicer Promoter
vector
p63
Relative luciferase activity
2.5
2.0
1.5
1.0
0.5
0.0
*
#
Vector
Nur77 ΔDBD
D
Input
IP: myc
GFP-p63 + + + + + + + + + +
myc-Nur77-WT - + + - - - + + - -
myc-Nur77 ΔDBD - - - + + - - - + +
Hypoxia + - + - + + - + - +
myc
GFP
Nur77-WT
Nur77ΔDBD
IgG
p63
IgG

Figure S6. Let-7i-5p inhibitor reverses Nur77 silencing induced reduction of tumor sphere formation and p110α decrease. (A) HCT116 cells was transfected with let-7i-5p inhibitor for 24 h under hypoxia, and expression of let-7i-5p was detected by qPCR. U6 was included as an internal control. Relative quantification was achieved by normalization to the amount of U6 using $2^{-\Delta\Delta Ct}$ method. Data is presented as mean ± SD. of three biological replicates**;** * indicates *P* < 0.05 in paired Student's *t* test (NS mimic compared with let-7i-5p mimic). **(B)** HCT116 or SW480 cells were co-transfected with Nur77 siRNA and let-7i-5p inhibitor for 24 h under hypoxia. Transfected cells were allowed to form tumor spheres. Spheres were photographed. **(C)** The p110α protein expression was determined by western blot. β-actin was included as an internal control. Data is presented as one of three independent experiments **(B)**, **(C).**

Figure S6

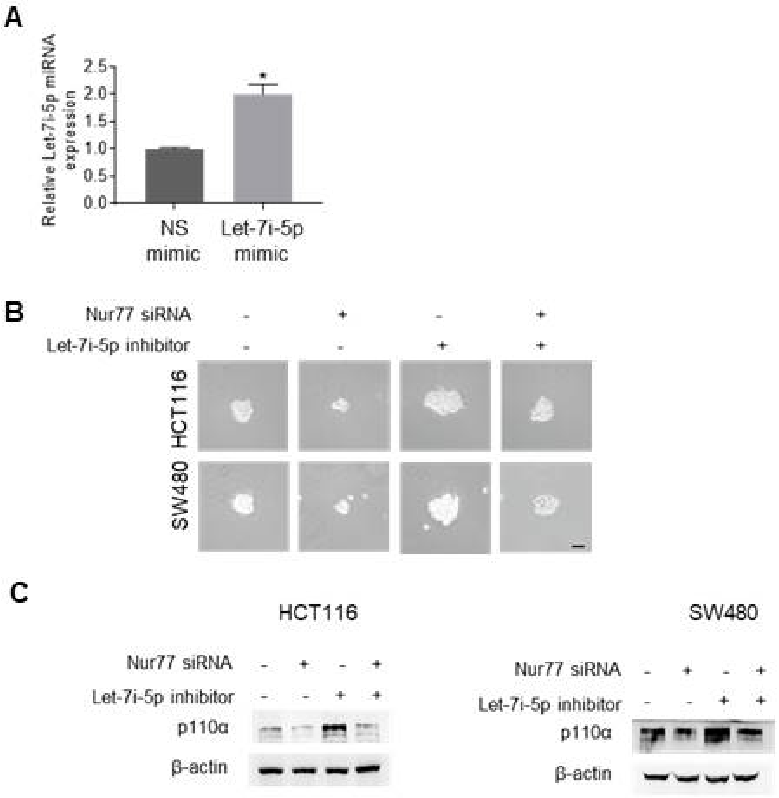
A
Relative Let-7i-5p miRNA expression
2.5
2.0
1.5
1.0
0.5
0.0
*
NS mimic
Let-7i-5p mimic
B
Nur77 siRNA
Let-7i-5p inhibitor
HCT116
SW480
C
HCT116
SW480
Nur77 siRNA
Let-7i-5p inhibitor
p110α
β-actin
